# Spintronics in Two-Dimensional Materials

**DOI:** 10.1007/s40820-020-00424-2

**Published:** 2020-04-18

**Authors:** Yanping Liu, Cheng Zeng, Jiahong Zhong, Junnan Ding, Zhiming M. Wang, Zongwen Liu

**Affiliations:** 1 School of Physics and Electronics, Hunan Key Laboratory for Super-Microstructure and Ultrafast Process, Central South University, 932 South Lushan Road, Changsha, 410083 Hunan People’s Republic of China; 2 Shenzhen Research Institute of Central South University, A510a, Virtual University Building, Southern District, High-Tech Industrial Park, Yuehai Street, Nanshan District, Shenzhen, People’s Republic of China; 3 State Key Laboratory of High-Performance Complex Manufacturing, Central South University, 932 South Lushan Road, Changsha, 410083 Hunan People’s Republic of China; 4 Institute of Fundamental and Frontier Sciences, University of Electronic Science and Technology of China, Chengdu, 610054 People’s Republic of China; 5 School of Chemical and Biomolecular Engineering, The University of Sydney, Sydney, NSW 2006 Australia

**Keywords:** Spintronics, 2D materials, TMDCs, Heterostructure, Proximity effect

## Abstract

Spintronics, exploiting the spin degree of electrons as the information vector, is an attractive field for implementing the beyond Complemetary metal-oxide-semiconductor (CMOS) devices. Recently, two-dimensional (2D) materials have been drawing tremendous attention in spintronics owing to their distinctive spin-dependent properties, such as the ultra-long spin relaxation time of graphene and the spin–valley locking of transition metal dichalcogenides. Moreover, the related heterostructures provide an unprecedented probability of combining the different characteristics via proximity effect, which could remedy the limitation of individual 2D materials. Hence, the proximity engineering has been growing extremely fast and has made significant achievements in the spin injection and manipulation. Nevertheless, there are still challenges toward practical application; for example, the mechanism of spin relaxation in 2D materials is unclear, and the high-efficiency spin gating is not yet achieved. In this review, we focus on 2D materials and related heterostructures to systematically summarize the progress of the spin injection, transport, manipulation, and application for information storage and processing. We also highlight the current challenges and future perspectives on the studies of spintronic devices based on 2D materials.

## Highlights

- The recent progress of spin injection, spin transport, spin manipulation, and application in 2D materials was summarized.
- The current challenges and outlook of future studies in spintronics based on 2D materials and related heterostructures were discussed.

## Graphical abstract

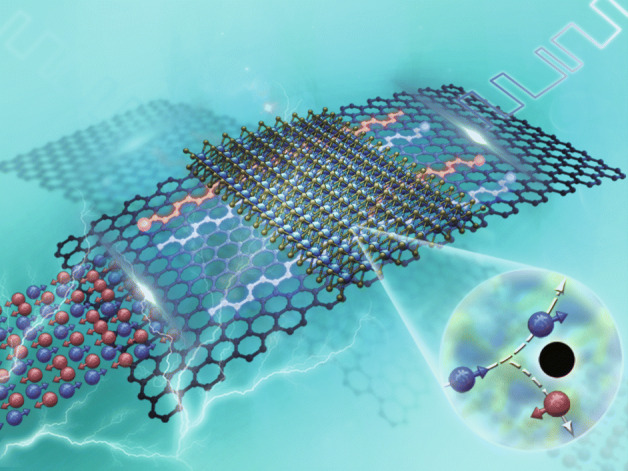

## Introduction

With the imminent end of Moore's law, exploiting new degrees of freedom has become an essential research direction to promote further development of electronic devices. The aim of spintronics is to utilize the spin degree of freedom of electrons to realize novel information storage and logic devices. A spintronic device has the superiority of faster speed, ultra-low heat dissipation, and non-volatility, making it an ideal candidate for future electronics. Additionally, 2D materials, such as graphene [1], black phosphorus (BP) [2], transition metal dichalcogenides (TMDCs) [3], and silicene [4], have created an excellent platform for spintronic research due to their unique spin-dependent properties, like ultra-long spin relaxation time and spin diffusion length, Rashba spin–orbit coupling (SOC), spin–valley locking, and quantum spin Hall effect. Furthermore, stacking individual 2D materials in a precisely designed order can combine the best of different components in one ultimate material [5, 6]. For example, the heterostructure of graphene and TMDCs enable graphene to have both excellent spin transport performance and larger SOC [7–9]. Along the way, 2D materials and related heterostructures can accomplish long-distance spin transport and effective spin manipulation, thereby realizing magnetic logic gates, magnetic random access memory (MRAM) [10, 11], and other spintronic devices.

However, several challenges remain to be solved in 2D material spintronics. Firstly, the 2D materials used in spintronics are mostly non-ferromagnetic [or the Curie temperature (*T*c) is far below room temperature]. Consequently, this requires a spin injection into the 2D materials by various methods, which brings a new issue on improving the polarization efficiency of the spin injection. On the other hand, efficiently manipulating the spin and maintaining the spin state are not yet achieved. The inability to transmit spin information and switch spin states means that the application of spin is impossible. Over the past few years, much of the effort has gone into seeking solutions to these topics and much progress has been made, such as the continuous improvement of spin parameters [12], the discovery and research of the novel 2D materials [13], the magnetic engineering of non-magnetic 2D materials [14], and the in-depth study of various spin effects. In this review, while we recapitulate the pioneering work of spintronics in 2D materials, we focus on the recent research and development in this area. The first section of this review presents the spin injection in 2D materials, while the second section reviews the research of spin transport in 2D materials. The third section describes the ways to manipulate spin, and the final section discusses the application of 2D materials in spintronic devices.

## Spin Injection in 2D Materials

Spin injection is a key and essential topic in the research and application of spintronics in 2D materials. A simple solution is to produce magnetism in 2D materials, thereby obtaining a spin-polarized state. Besides, many other approaches to inject spin have been proposed, including electrical injection, optical injection, and spin–orbit coupling effect.

### Magnetic Engineering of Non-magnetic 2D Materials

At present, the widely used 2D materials in spintronic research, such as graphene and TMDCs, are non-magnetic. Therefore, magnetic engineering is a significant topic to obtain spin-polarized states in 2D materials, especially through gating, doping, functionalization. Magnetism originates from the moving charges and spin of elementary particles and is commonly deemed unstable in 2D systems on account of fundamental hindrances, such as thermal disturbance. Through the efforts of recent years, it is now possible to realize long-range magnetism in 2D systems and many achievements have been made in the magnetic engineering of non-magnetic 2D materials.

**Fig. 1:**
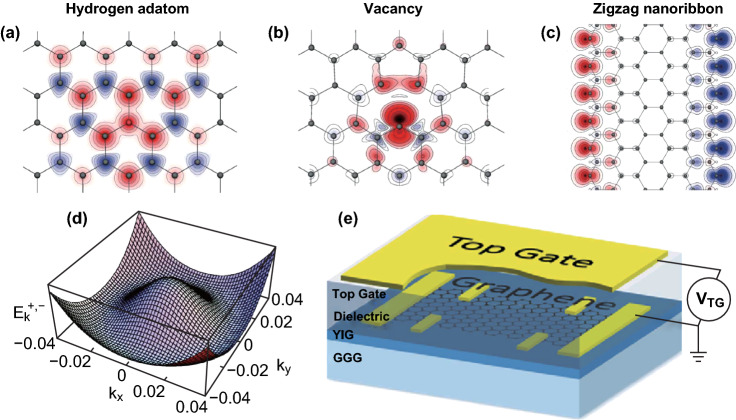
Schematic diagram of magnetic engineering strategy. **a** Hydrogenated graphene. **b** Vacancy graphene. **c** Graphene nanoribbon. Different colors represent magnetic moments in different directions [15]. **d** Calculated Mexican hat band dispersion in electrically biased Bernal stacked bilayer graphene. The diverging electronic DOS at the edge of the Mexican hat potentially results in ferromagnetic Stoner instability [38]. **e** Schematic drawing (with top gate) of the devices, which can prove the proximity-induced ferromagnetism in graphene transferred to a YIG/GGG substrate [41]. Figures reproduced with permission from Refs. [15, 38, 41]. (Color figure online)

The mainstream strategy is through introducing vacancies or adding adatoms in 2D materials [14] that uses unpaired electrons to obtain local magnetic moments [15], such as hydrogenated graphene [16–19] (Fig. 1a), vacancy graphene [16, 20, 21] (Fig. 1b), and graphene nanoribbons [22–24] (Fig. 1c). As shown in Fig. 1a, hydrogen chemisorbs reversibly on graphene, forming a strong covalent bond [25]. This effectively removes one $P_{{\mathrm{Z}}}$ orbit (it shifts the bonding state down by several electron volts) from the $\pi$ band, thus creating a sublattice imbalance [15]. And a single H atom leads to a quasi-localized state with a magnetic moment of 1 $\mu_{{\mathrm{B}}}$. Also, as an analogue of fully hydrogenated graphene but far more complex, graphane has been noticed and predicted to possess unique properties [26]. Moreover, organic molecules could change the properties of graphene [27] and graphane (CH), [28] by absorbing on the surface. However, the magnetic states may depend on the type [29–31], concentration [16, 19, 29], and distribution [16, 32, 33] of the adsorbates, as well as the stacking order in case of multilayer graphene [11, 34]. A detailed summary of the theoretical calculations under different adsorption conditions can be found in Ref. [11]. Besides, by removing four electrons from the bands, a single vacancy in graphene generates a local spin-polarized electronic density, which could be further connected by cruise electrons, as shown in Fig. 1b. However, realizing long-range ferromagnetic order is still a considerable challenge through the above methods, and even there was a report [20] questioned the feasibility of these approaches, because only the paramagnetic response in graphene with fluorine adatoms or vacancies at low temperatures has been observed. What’s more, there was only limited experimental work on magnetic engineering, and most of the results were theoretically calculated based on assumptions. In reality, 2D materials are normally supported by substrates, which may affect the results [18, 35]. Although there have been many reports on the formation of ferromagnetism in non-magnetic 2D materials, no consensus has been reached. The main argument has been focused on the source of the detected signal, which might be from the ferromagnetic impurities of the substrate [36, 37].

Additionally, graphene nanoribbons have extended defects, which result in dangling bonds formed by unpaired electrons at the boundary, contributing to magnetic moments, as shown in Fig. 1c. Nonetheless, these chemically active extended defects are easily passivated, giving rise to unstable magnetic properties. Also, theoretically, strict long-range 1D ferromagnetic order cannot exist [22]. Band structure engineering is a vital route to create 2D ferromagnetism without the assistance of structural imperfections, which was predicted to exist in electrically biased bilayer graphene [38, 39] and doped GaSe [40]. In biased bilayer graphene, the vertical electric field causes the electrostatic potentials of the two layers different, opening a band gap and inducing a Mexican hat distribution (Fig. 1d) at low energy, which is usually accompanied by the itinerant ferromagnetism. However, experimentally, the ferromagnetism has not been confirmed yet. It was speculated that this might be due to the limited total magnetic moments or low $T_{{\mathrm{C}}}$. Similarly, the doped GaSe should also display the Mexican hat distribution.

Recently, there have been a large number of reports on proximity effect, which provides non-magnetic 2D materials with a solution of borrowing magnetic properties from neighboring magnetic materials, especially ferromagnetic insulators. Conversely, 2D materials provide a unique platform for exploring the full potential of magnetic proximity effect. Through transferring the exfoliated graphene to ferromagnetic insulator yttrium ion garnet (YIG, Fig. 1e) [41] or EuS [42], a spin precession, caused by a sufficiently strong exchange field, can be observed, which is significant evidence of the presence of ferromagnetism in graphene. Also, the magnetic proximity coupling can significantly lift the valley splitting in WS_2_ by first-principles calculation that is valuable to achieve spin polarization [43]. Also, if the magnetic insulator is a 2D material, a seamlessly integrated and interacting van der Waals (vdW) heterostructure can be formed, and the strong atomicity at the interface is favorable for interfacial exchange.

### Electrical Injection in 2D Materials

Though magnetic engineering can induce spin-polarized states and thus acquire magnetism in 2D materials, electrical injection is a more common and practical strategy to produce spin-polarized states for spintronic devices. It was theoretically predicted that the spin injection efficiency (illustrated by spin injection polarization, $P = (N_{ \uparrow } - N_{ \downarrow } )/(N_{ \uparrow } + N_{ \downarrow } )$) from ferromagnetic electrodes into graphene can be as high as 60–80% [44]. In practice, when polarized electrons are directly injected into a 2D material through a ferromagnetic electrode, due to conductivity mismatch, spin injection efficiency is extremely low [45–51]. The spin injection polarization can be described by Eq. 1 [48]:

1 $$P = \frac{{r_{{\mathrm{F}}} P_{{\mathrm{F}}} + r_{{\mathrm{C}}} P_{{\mathrm{C}}} }}{{r_{{{\mathrm{FN}}}} }}$$

with the total effective resistance $r_{{{\mathrm{FN}}}} = r_{{\mathrm{F}}} + r_{{\mathrm{N}}} + r_{{\mathrm{C}}}$, in which $r_{{\mathrm{F}}}$, $r_{{\mathrm{N}}}$, $r_{{\mathrm{C}}}$ are the effective resistance of the ferromagnetic electrode, 2D material, contact, respectively. $P_{{\mathrm{F}}} = \left( {\sigma_{ \uparrow } - \sigma_{ \downarrow } } \right)/\left( {\sigma_{ \uparrow } + \sigma_{ \downarrow } } \right)$ is the conductivity polarization of the ferromagnetic electrode, and $P_{{\mathrm{C}}}$ is the conductivity polarization of the contact. For direct contact ($r_{{\mathrm{C}}} = 0$), because of *r*_F_ ≪ *r*_N_, $P$ is very low.

**Fig. 2:**
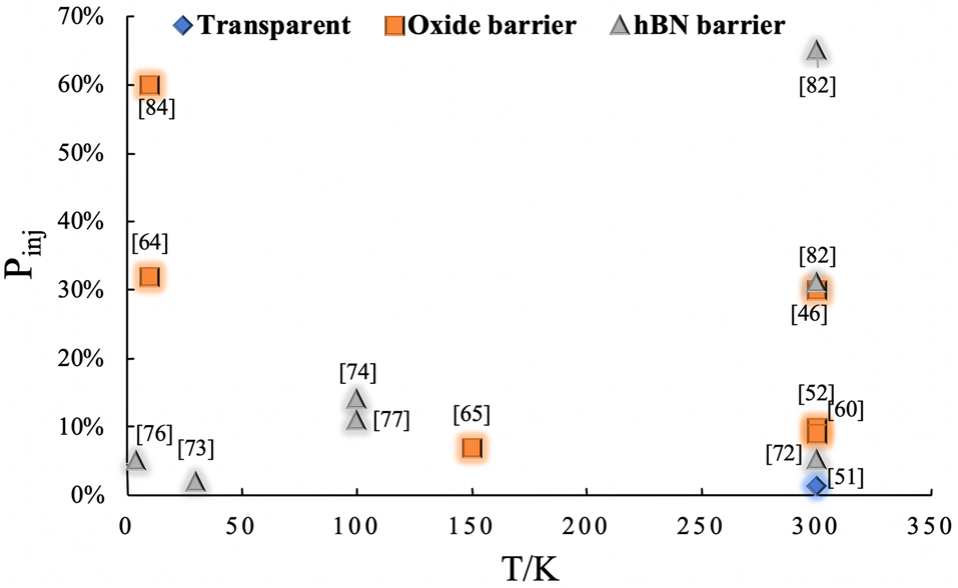
Development of electrical injection with different tunnel barriers. P_inj_ stands for the spin injection polarization in graphene. And *T* shows the experimental temperature. The numbers are the relevant reference. It should be noted that the above statistics are incomplete, just representative results

To solve the conductivity mismatch and improve the spin injection efficiency, a tunneling injection method was proposed, in which a tunnel barrier was added between the ferromagnetic electrode and the 2D material ($r_{{\mathrm{C}}} \ne 0$) [45, 46, 48, 49, 52–66]. Generally, the tunnel barrier is a metal oxide, such as MgO, Al_2_O_3,_ and TiO_2_. However, growing a layer of oxide of an appropriate thickness and defect-free (with electron tunneling and no backflow) became a new challenge [45, 52, 53, 62, 63]. Related research has attempted to solve these problems and achieved positive results by improving growth techniques [53, 62, 63, 67] or incorporating other materials [45, 46]. For example, if the vdW 2D insulator is served as a tunnel barrier, such as hBN [68], the spin depolarization caused by interface defects can be well solved. Comparison of some representative results is shown in Fig. 2. This is because the surface of hBN is atomically smooth, and has few charge inhomogeneities [69]. Furthermore, atomically hBN is an isomorph of graphite with a similar hexagonal layered structure and a small lattice mismatch [70] of ~ 1.8%, exerting less strain on graphene [71]. Therefore, there is less surface state at the interface and the charges or spin traps are minimized.

Surprisingly, theoretical calculation shows that the spin injection efficiency can reach 100% with the increase in the hBN’s thickness by using a Ni electrode [68], which has a good lattice match with both the graphene and the hBN. Experimentally, both of exfoliated hBN [72, 73] and CVD-hBN [74–77] have been extensively studied as a tunnel barrier. What’s more, it was indicated that a thicker hBN tunneling layer could achieve higher spin polarization [78]. For instance, Singh et al. experimentally demonstrated that bilayer hBN is a better choice for tunnel barrier than monolayer hBN [72]. And recently, Leutenantsmeyer et al. have characterized the spin injection into bilayer graphene with a trilayer hBN tunnel barrier and compared the result with that of the spin injection achieved with bilayer hBN [79].

**Fig. 3:**
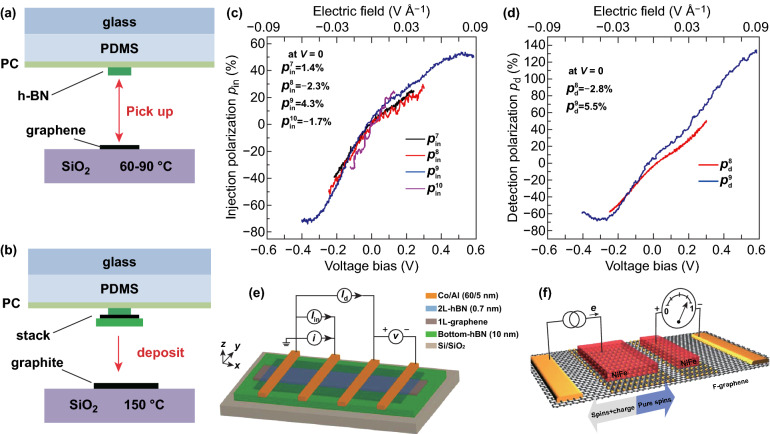
**a, b** Diagram of the fast pickup technique. **a** Picking up process. **b** Depositing process [80]. **c–e** Experiment of bias-induced differential spin injection ($p_{{{\mathrm{in}}}}$) and detection ($p_{{\mathrm{d}}}$) polarizations. **c** $p_{{{\mathrm{in}}}}$ of four contacts, as a function of the DC voltage bias *V*. **d** $p_{{\mathrm{d}}}$ for two different detector contacts, as a function of DC voltage bias *V* applied across the detector, while the injector bias is fixed at $I_{{{\mathrm{in}}}} = + 20\,\upmu {\mathrm{A}}$. **e** Schematic diagram of measuring device structure [83]. **f** Schematic image of the device in which the fluorinated graphene serves as a tunnel barrier [84]. Figures reproduced with permission from Refs. [80, 83, 84]

Generally, the graphene/hBN heterostructure is fabricated by the transfer technology. Although the transfer technology comes in various forms, the fast pickup technique [80] (Fig. 3a, b) is relatively mature [81], and the possible residue on the material interface is considered the source for the great disparity between the experimental value and the theoretical prediction. On this account, growing hBN directly on graphene by CVD may be a viable solution and the annealing is of great importance to improve the device performance. Moreover, a bias can further increase the spin injection polarization. Kamalakar et al. made the enhanced magnitude of the spin polarization up by ~ 65%, which was an order of magnitude higher than the result achieved with oxide barriers and much higher than the maximum intrinsic spin polarization of Co (~ 35%), through the changing of the CVD-hBN thickness and the resistance of Co/CVD-hBN/graphene interface. Meanwhile, they observed the signal inversion in graphene for the first time [82]. In another report, unexpected large and bias-induced (differential) spin injection (detection) polarizations close to ± 100% (Fig. 3c, d) have been discovered in FM/bilayer hBN/graphene/hBN heterostructures [83], as shown in Fig. 3e. The authors also found a distinction between the exfoliated hBN and the CVD-hBN, which might result from stacked layers (refer to Fig. 3c).

Separately, Friedman et al. reported a very high spin injection efficiency ($> 60\%$) with a graphene bilayer in which the fluorinated upper layer served as a tunnel barrier and the non-fluorinated bottom layer acted as a high-mobility transport channel [11, 84], as shown in Fig. 3f. Indeed, through the control of the graphene/FM interface area to enhance the contact resistance, it can realize high-efficiency spin injection and detection in the case of transparent contact (without the tunnel barrier) [85].

In addition, 2D ferromagnetic materials can effectively enhance the spin injection polarization. For example, it was found that CrI_3_ as a tunnel barrier, tunneling electrons could be scattered multiple times across the alternatively spin-polarized layers, resulting in large spin polarizability [86–89]. This result was attributed to the magnon-mediated tunneling process (with ferromagnetic barriers, CrI_3_ [89], CrBr_3_ [90]), in contrast to the conventional phonon- or electron-mediated tunneling (with non-magnetic barriers). On the other hand, 2D ferromagnetic materials, such as Fe_3_GeTe_2_, can serve as a ferromagnetic electrode. Due to better interface contact between 2D materials, enhanced spin injection polarization may be obtained. Additionally, half-metal materials with high spin polarization, such as Heusler alloys [91] and topological insulators (TI) [92], are now good candidates for achieving high spin injection polarization. Similarly, 2D magnetic half-metal materials are also worthy of attention. Possible 2D half-metals predicted by theory include manganese trihalides [93], FeCl_2_, FeBr_2_, FeI_2_ [94], Janus structure of monolayer MnSSe [95], and 2H-VSe_2_ [96]. However, currently found 2D ferromagnetic materials in room temperature only include VSe_2_ [97], MnSe_2_ [98], and gate-tuned Fe_3_GeTe_2_ [99]. The ferromagnetic phase transition temperature ($T_{{\mathrm{C}}}$) of almost all other magnetic 2D materials is still much lower than the room temperature (refer to Table 1). Hence, for practical application and research, a great effort needs to be directed toward high-temperature 2D magnets. While this is a huge challenge, the potential of realizing high-temperature ferromagnetic behavior in 2D materials is attractive. Moreover, the fundamental restriction of 2D long-range magnetic order is nonexistent theoretically, but the enhanced thermal fluctuation is rather always a hindrance. Based on the available research information, the rule of thumb in designing high-temperature 2D ferromagnetic materials is to strengthen the exchange interaction and the uniaxial magnetic anisotropy [14].

**Table 1:** Part of the 2D magnetic materials

2D material	Tc	Electric properties	Magnetic properties
Cr_2_Ge_2_Te_6_	30 K (bilayer) 68 K (bulk) Weak magnetic dependence	Insulator	Ferromagnetism
Cr_2_Si_2_Te_6_	33 K (monolayer > bulk) 290 K (under stress)	Semiconductor	Ferromagnetism
Fe_3_GeTe_2_	205 K (bulk) 150–220 K (Fe occupancy) 300 K (monolayer, gate tune)	Metal	Ferromagnetism
VSe_2_-1T	300 K (monolayer)	Metal	Ferromagnetism (monolayer, controversial) Paramagnetic (bulk)
VS_2_-1T	300 K (few layers)	Metal	Ferromagnetism (few layers) Ferromagnetism (monolayer, theoretic)
MnSe_2_-1T	300 K (monolayer)	Metal	Ferromagnetism (ultra-thin layer) Antiferromagnetism (bulk)
CrI_3_	45 K (monolayer) 61 K (bulk)	Insulator	Ferromagnetism
CrBr_3_	37 K (monolayer)	Insulator	Ferromagnetism
ReI_3_	65 K (theoretic)	Half-metal	Ferromagnetism (theoretic)
ReBr_3_	390 K (theoretic)	Half-metal	Ferromagnetism (theoretic)

### Optical Injection in 2D Materials

During electrical injection, the contact between the spin transport channel and the electrode (or the tunnel barrier) causes spin-dependent scattering at the interface, which affects the efficiency of spin injection and limits the potential of materials in spintronic research. Instead, the nondestructive optical injection has no such drawback. In the presence of a strong SOC, the interaction between the incident light and the orbital degree of freedom of graphene makes a possible spin injection. Inglot et al. reported that by linearly polarized incident light, without any FM electrode, direct injection of spin-polarized current into graphene could be achieved. The SOC of graphene comes from the substrate-induced Rashba effect and is assisted by external static magnetic fields in the plane. Furthermore, by adjusting the intensity of the SOC and the magnetic field, up to 22% of the spin injection polarization can be achieved [100]. Besides, without the assistance of the in-plane magnetic field, Rioux and Burkard have also realized optical injection by linearly polarized incident light. And through controlling the gate voltage and the incident light frequency, spin polarization could reach up to 75% [101].

**Fig. 4:**
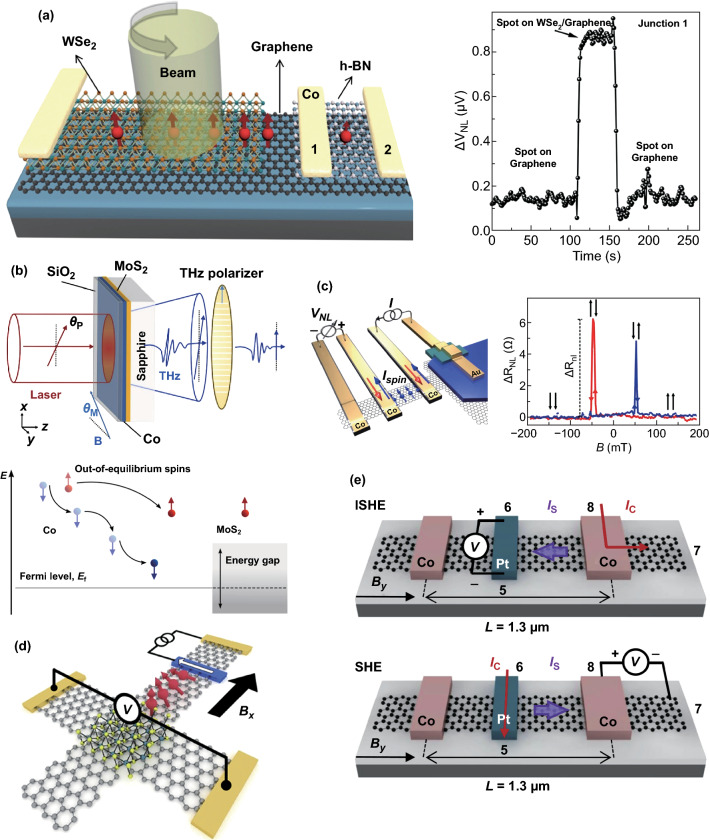
**a** Schematic of the optical injection structure with WSe_2_. Utilizing circularly polarized light, the spin-polarized electrons will generate in WSe_2_, and then diffuse into graphene and cause non-local spin signals, as shown on the right. When there is no light, the signal is in its initial state. And once there is light, the signal rises rapidly [104]. **b** Schematic of Co/MoS_2_ heterostructure and experimental configuration. The figure below describes the principle of spin injection [105]. **c** Schematic of Bi_2_Te_2_Se/graphene heterostructure spin injection and the non-local spin signals [85]. **d** Schematic of spin Hall effect in graphene with MoS_2_ at room temperature [119]. **e** Spin-to-charge conversion in a trilayer graphene/Pt lateral heterostructure. Sketch of the inverse spin Hall effect (top) and spin Hall effect (bottom) measurement configurations [120]. Figures reproduced with permission from Refs. [85, 104, 105, 119, 120]

On the other hand, the spin–valley coupling of TMDCs provides an attractive approach for optical injection and derives a novel field called valleytronics [102]. The valley selectively absorbs the circularly polarized light, which can excite spin-polarized carriers, and those spin-polarized carriers can diffuse into the neighboring graphene layer, realizing high-efficiency spin injection. For example, Luo et al. fabricated monolayer MoS_2_/few-layer graphene hybrid spin valves and demonstrated, for the first time, the opto-valleytronics spin injection and lateral spin transport in room temperature [103]. Avsar et al. achieved spin injection by using monolayer WSe_2_/graphene and utilized polarization-dependent measurement to prove that the spin originated from the monolayer WSe_2_ [104], as shown in Fig. 4a. They then conducted research of bilayer WSe_2_ and found that spin polarization was absent because of the restored inversion symmetry in bilayer WSe_2_, which further confirmed that the spin-polarized current in graphene was derived from the optical injection.

Recently, Cheng et al. [105] have taken advantage of a femtosecond laser to form far out-of-equilibrium spin populations in a Co electrode, and successfully injected spin-polarized current directly into a monolayer MoS_2_ semiconductor (Fig. 4b). They also estimated the magnitude of spin current density to attain 10^6^–10^8^ A cm^−2^, which is a orders of magnitude larger than the amplitudes of a typical spin current injection into a semiconductor (for example, 10^2^–10^3^ A cm^−2^ using spin pump [106] and 10^–1^–10^0^ A cm^−2^ via tunnel junction [107, 108]). With the incident femtosecond laser normal on Co, the far out-of-equilibrium spin population was generated in ferromagnetic Co. As the excited carriers diffused, they relaxed in energy. Moreover, due to the strong spin asymmetry in Co, a significant number of majority spin electrons would persist for a longer time at high energies. As theoretically proposed [109], only the majority spin electrons with energy higher than the semiconductor conduction band minimum could cross into the semiconducting layer [105], as shown in the bottom of Fig. 4b.

### SOC Effects in 2D Materials

Spin–orbit coupling (SOC) is the relativistic interaction between the spin and momentum degrees of freedom of electrons. In low dimensions, the SOC effects are greatly enhanced, and the new phases of matter, such as spin-polarized surface and interface states, get emerged. At the surface or interface, inversion symmetry is broken and the resultant electric field couples with the spin of itinerant electrons, generating spin splitting, known as Rashba SOC, with the corresponding Hamiltonian,

2 $${\text{H}}_{{\mathrm{R}}} = {\text{v}}_{0} {\hat{\mathbf{z}}} \cdot \left( {{\mathbf{k}} \times {{\varvec{\upsigma}}}} \right)$$

where $v_{0}$ is the Rashba parameter and ${\hat{\mathbf{z}}}$ is the unit normal to the surface or interface, while ${\mathbf{k}}$ is momentum, and ${{\varvec{\upsigma}}}$ is the spin. Moreover, (2) corresponds to an effective ${\mathbf{k}}$-dependent magnetic field,

3 $${\mathbf{B}}\left( {\mathbf{k}} \right) = 2\alpha {\hat{\mathbf{z}}} \times {\mathbf{k}}$$

The coupling parameter $\alpha$ depends on the potential and the external field that may be applied by gates. Besides, spin polarization also exists on the surface of TI, with additional topology properties (The Hamiltonian has the same Rashba form). In both cases, a strong 2D SOC locks the electron spin and momentum together, directly affecting the interaction between the charge and spin transport. These low-dimensional SOC-based effects are generally robust and can be explored at room temperature [110]. Hence, the SOC-based effects have a great significance to realize all-electric spintronic devices.

**Fig. 5:**
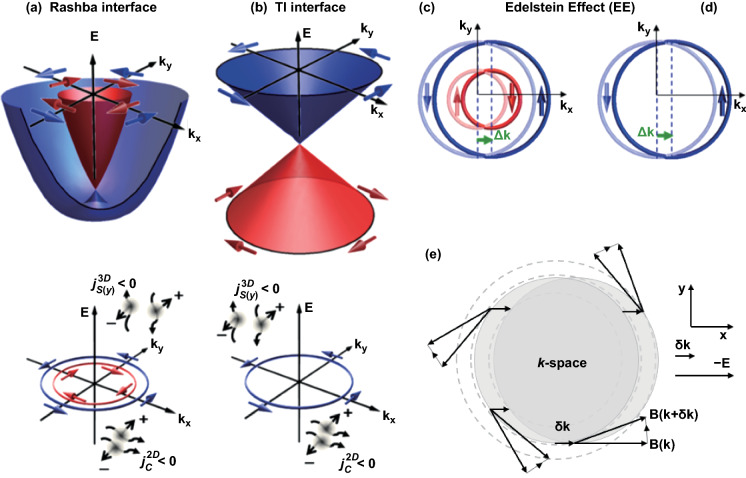
Edelstein effect. Top: **a** Energy dispersion surfaces of the 2D states at a Rashba interface and **b** Dirac dispersion cone of the surface or interface states of a topological insulator. Bottom: **a** Fermi contours of Rashba states with two contours with helical spin configurations of opposite chirality and **b** TI surface or interface states. **c**, **d** Edelstein effect: A flow of electrons along *x* ($j_{{\mathrm{c}}}^{2D\left( x \right)} < 0$ in the figure) in **c** Rashba or **d** TI. 2DEGs are associated with shifts $\Delta k$ of the Fermi contours generating an extra population of spin along the direction. (For Rashba, there is a partial compensation of the contributions of the two contours.) **e** Schematic picture of intrinsic spin–orbit generated spin currents. An electric field in the *x*-direction displaces the Fermi distribution by $\delta k$. Carriers experience a torque that tilts them according to their spins. The tilting is opposite for opposite momenta and it generates a spin current in the *y*-direction. Figures reprinted with permission from Ref. [113]

According to Edelstein or inverse Edelstein effects [111] (Fig. 5), Rashba surface and topological surface states with spiral spin polarization distribution at Fermi surface can realize conversion between the spin current and the charge current. The spin Hall effect of heavy metals is another type of conversion by SOC effects in 3D [112]. As shown schematically in Fig. 5e [113], an in-plane charge current can shift the Fermi contours in the direction of the current (x-direction). The electric field changes ${\mathbf{k}}$ and forces the electrons out of alignment with ${\mathbf{B}}\left( {\mathbf{k}} \right)$. Therefore, while moving in the momentum space, the electrons experience an effective torque which tilts the spins, and the spin tilt in opposing directions on opposite sides of the Fermi surface (${\mathbf{k}}_{{\mathbf{y}}} > 0\,\, {\text {or}} < 0$). The spin accumulation will eventually occur perpendicular to the current direction in the plane (y-direction). Furthermore, the accumulated spins can diffuse into the adjacent layer, resulting in a pure 3D spin current. There are some predicted 2D TIs of first-principles calculations that are worth noting, including the α-tin [114, 115], dumbbell tin [116], silicene/germanene [117] and the decorated Bi/Sb [118].

Experimentally, Vaklinova et al. demonstrated the injection of spin-polarized current into graphene (Fig. 4c) from a topological insulator, Bi_2_Te_2_Se, whose 2D surface states host massless Dirac Fermions. In several sets of the experiments, the highest spin injection polarization reached 10%. Although there was doubt about the source of the spin signals, which might come from the surface state of TI or from spin Hall effect caused by the proximity effect, the authors analyzed the signal source according to the trend of non-local spin signal with various conditions and were able to basically determine that the signal originated from the surface states of TI [85]. Moreover, the non-local spin signal was detected with a transparent contact in this experiment. In addition, by combining Hall probes with ferromagnetic electrodes (Fig. 4d) and varying temperatures up to room temperature [119], Safeer et al. unambiguously demonstrated experimentally the spin Hall effect in graphene induced by MoS_2_ proximity. Furthermore, theoretical calculation shows that WS_2_ can maximize the spin proximity effect in graphene compared to graphene on MoS_2_, WSe_2_, or MoSe_2_. To enhance the spin proximity effect, the highest interface quality should be sought [9]. As a heavy metal, Pt has a strong SOC similar to MoS_2_, and it has been reported that the spin Hall effect in Pt could generate pure spin currents in a few-layer graphene channel at room temperature [120], as shown in Fig. 4e.

## Spin Transport in 2D Materials

While the implementation of spin injection in materials has been successful, an elementary issue in spintronics is how to maintain the spin states for a long enough time and a long enough distance to complete the transmission of information. Take that into consideration, 2D material is an ideal platform with great advantages.

**Fig. 6:**
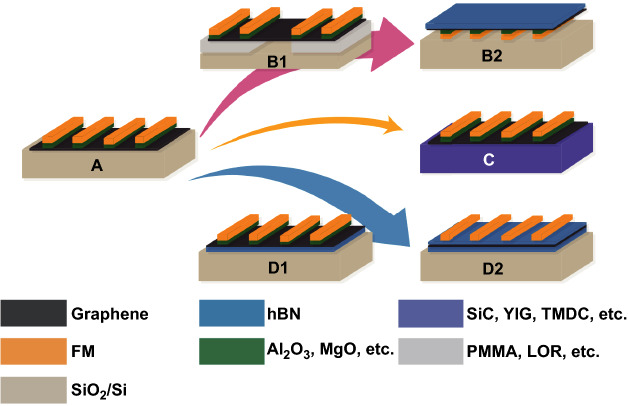
Development trend of spin device structure (graphene as an example). **A** Non-local spin valve with oxide tunnel barriers, **B** with suspended graphene, **C** with a different substrate, **D** with hBN as encapsulation or tunnel barrier

**Fig. 7:**
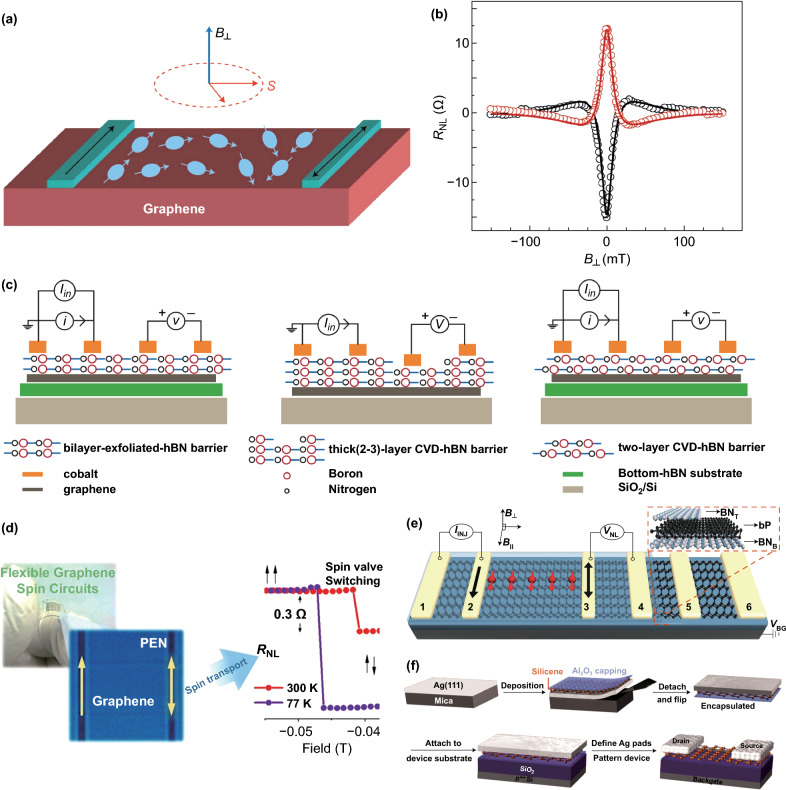
**a, b** Schematic of Hanle measurement. Non-local spin valve structure. Spin-polarized electrons, generated by FM, diffuse through graphene channel and form pure spin current. When the vertical magnetic field is added, the electron’s spin will process around the field and a classical Hanle signal can be obtained by scanning the magnetic field [15]. **c** Comparison diagram of the different hBN as a tunnel barrier [125]. **d** Schematic of flexible graphene spin circuits [142]. **e** Non-local spin valves structure with BP [154]. **f** Schematics of silicene FET and its synthesis–transfer–fabrication process, which includes the following key steps: epitaxial growth of silicene on crystallized Ag(111) thin film, in situ Al_2_O_3_ capping, encapsulated delamination transfer of silicene, and native contact electrons formation to enable back-gated silicene transistors [160]. Figures reproduced with permission from Refs. [15, 125, 142, 154, 160]

2D materials, such as graphene, BP, and silicene, are the excellent channels of spin and the crucial materials for the practical application of spin devices. Theoretical estimations indicate that in 2D materials, the spin diffusion length can be as long as ~ 10 μm and the spin relaxation time is up to ~ 1 μs at room temperature [15], which are several orders of magnitude larger than the traditional metal conductors. Emphatically, for the research of spin transport, the non-local spin valve is still the most vital structure. The usual non-local spin valve is a four-electrode structure, with two ferromagnetic electrodes in the middle as the injector and detector, and a 2D material channel at the bottom. Although many innovative structures have emerged in the research (refer to Fig. 6 and Table 2), the fundamental has not changed yet. Moreover, the non-local spin valve can be used for Hanle measurements (Fig. 7a, b), which is able to demonstrate spin transport and calculate the spin relaxation time ($\tau_{{\mathrm{s}}}$) and the spin diffusion length ($\lambda_{{\mathrm{s}}} = \sqrt {\tau_{{\mathrm{s}}} D_{{\mathrm{s}}} }$, where $D_{{\mathrm{s}}}$ is the diffusion coefficient) by fitting the data with Eq. 4 [15],

4 $$R_{{{\mathrm{NL}}}} \propto \pm \mathop \int \limits_{0}^{\infty } \frac{1}{{\sqrt {4\pi D_{{\mathrm{s}}} t} }}\exp \left[ { - \frac{{L^{2} }}{{4D_{{\mathrm{s}}} t}}} \right]\cos \left( {\omega_{{\mathrm{L}}} t} \right)\exp \left( { - {\raise0.7ex\hbox{$t$} \!\mathord{\left/ {\vphantom {t {\tau_{{\mathrm{s}}} }}}\right.\kern-\nulldelimiterspace} \!\lower0.7ex\hbox{${\tau_{{\mathrm{s}}} }$}}} \right){\text{d}}t$$

**Table 2:** Development of spin transport research

Year	Structure	$\tau_{{\mathrm{s}}} {\text{/ns}}$	$\lambda_{{\mathrm{s}}} {/}\upmu {\text{m}}$	*T*/K	References
2007	**SiO**_**2**_**/Si (sub)**	**0.17**	**2**	**300**	[52]
2012	**SiC (sub)**	**1.3**	**0.56**	**300**	[130]
2012	**hBN (sub)**	**0.39**	**4.5**	**300**	[137]
2014	**hBN (sub) and hBN-encapsulated**	**2**	**12**	**300**	[140]
2016	hBN (sub) and ML-hBN-encapsulated*	**0.18**	**5.1**	**300**	[141]
2016	hBN (sub) and 2–3L-hBN-encapsulated*	**1.86**	**5.79**	**300**	[72]
2016	**Suspension and hBN-encapsulated**	**12.6**	**30.5**	**300**	[147]
2019	**Flexible substrate**	**0.25**	**8**	**300**	[142]
*2017*	hBN (sub) and hBN-encapsulated*	*4*	*6*	*100*	[154]
*2017*	***SiO***_***2***_***/Si (sub)***	***46***	***0.24***	***12***	[161]

Bold, graphene; italic, BP; bold–italics, MoS_2_. It should be noted that the below statistics are incomplete, just representative results. *means that the encapsulated hBN is also a tunnel barrier

where the $\pm$ represents the parallel or antiparallel magnetization state, and $L$ is the distance from the injector to the detector, while $\omega_{{\mathrm{L}}} = g\mu_{{\mathrm{B}}} B_{ \bot } /\hbar$ ($\mu_{{\mathrm{B}}}$ is the Bohr magneton and $\hbar$ is reduced Planck constant).

Figure 6 shows that graphene was extensively studied in spin transport. The ultra-high carrier mobility and weak intrinsic spin–orbit coupling of graphene make the electron spin be carried nearly unaffected over unprecedented distances, even at room temperature [15, 121–124]. Initially, the research of spin transport was performed in graphene samples on a silicon substrate prepared by exfoliation or CVD growth (Fig. 6A). Tombros et al. reported the spin transport in single graphene with a silicon substrate at room temperature for the first time. They obtained the highest spin relaxation time of ~ 170 ps and a spin diffusion length of ~ 2 μm [52]. However, early studies on the silicon substrate did not achieve a spin relaxation time of more than 500 ps and mobility of more than 5000 cm^2^ V^−1^ s^−1^, which are several orders of magnitude lower than expected [45, 125]. Such low values are believed to be caused by residual impurities during the fabrication of the device and the scattering of electrical and adsorbed atoms on the silicon substrate [126–129].

To improve the spin relaxation time and the spin diffusion length, there have been attempts to epitaxially grow graphene on other substrate materials, such as SiC [130, 131], YIG [132, 133], and TMDCs [8, 134, 135] (Fig. 6C). With the SiC substrate, the spin relaxation time of monolayer graphene at room temperature reached up to 1.3 ns [130], while the localized states in SiC were found to influence the spin diffusion transport through the interlayer hopping mechanisms [136]. Another approach is to use hBN as a substrate, and transfer graphene into the hBN (Fig. 6D1). Zomer et al. [137] fabricated the first graphene spin valves on an hBN substrate and showed high mobility of ~ 40,000 cm^2^ V^−1^ s^−1^ and enhanced spin diffusion length up to ~ 4.5 μm. But the spin relaxation time was only ~ 390 ps, which is similar to the value achieved on a silicon substrate. It was further demonstrated that there was no strong correlation between the spin relaxation time and the mobility of the graphene, which was consistent with a study of spin transport in graphene with different mobilities [138]. Also, it was shown that the charge scattering was not a major role in the spin relaxation time. Thus, more attention was paid to the effect of the residual solution on spin relaxation time during device fabrication [139], and an approach for hBN-encapsulated graphene was proposed (Fig. 6D2). It was shown [140] that a partial fully encapsulated monolayer graphene device could achieve a spin relaxation time of 2 ns and a spin diffusion length of 12 μm. Meanwhile, as a comparison, the measured spin relaxation time was only 0.3 ns in the incompletely encapsulated part. Additionally, Gurram et al. [141] fabricated a fully encapsulated monolayer graphene device with a top monolayer hBN, but obtained a spin relaxation time of only 176 ps and a spin diffusion length of 5.1 μm. The reason could be that the interface resistance of the monolayer graphene/hBN was extremely low, leading to unsatisfactory injection efficiency, while the hBN was too thin to protect graphene from contamination. Therefore, recently, they have encapsulated the graphene with bilayer hBN, which consisted of two individually stacked CVD hBN monolayers via the conventional wet transfer method [75], but only achieved a mobility below 3400 cm^2^ V^−1^ s^−1^ and a spin relaxation time below 400 ps. Such low values might be caused by solution residues during the transfer process (through the wet transfer method), or by a misalignment between unnaturally grown double-layered hBN (refer to Fig. 7c). Similarly, with hBN as a tunnel barrier, Singh et al. reported the spin relaxation time that exceeded a nanosecond (1.86 ns) at room temperature for the first time. Furthermore, they explored different layers of hBN and suggested 2–3 layers as optimal [72]. As can be seen from the above discussion, even though the same device structure was adopted, the results of Gurram and Singh are different, which was most likely due to the different stack forms of hBN (refer to Fig. 7c).

Moreover, Serrano et al. studied the spin transport on flexible substrates with a CVD monolayer graphene, as shown in Fig. 7d. Although on a rougher substrate, the spin diffusion coefficient still reached ~ 0.2 m^2^ s^−1^ at room temperature in long graphene channels (up to 15 μm). Compared to the Si/SiO_2_ substrate, such values were up to 20 times larger, leading to spin signals one order higher and an enhanced spin diffusion length of ~ 10 μm. In general, the intrinsic roughness of the polymer substrate, which is 4–5 times higher than the roughness of the standard silicon substrates, is not encouraging for spin transport compared to the Si/SiO_2_ substrate, but the carrier mobility was up to 10 times larger. Atomic force microscope (AFM) revealed that in the polymer, despite a higher roughness, the roughness is also wider, implying a reduction in protruding scattering peaks per unit area in comparison with a Si/SiO_2_ substrate (estimated reduction of up to 90%) [142].

In addition to the above methods, suspending graphene is a fantastic way to solve the substrate scattering (Fig. 6B). In this way, it is able to avoid the coupling of graphene and substrate electrons and so to avoid masking the intrinsic properties of 2D materials. However, initially, the most common technique to suspend graphene flakes was acid-based [143, 144], which was used to etch the substrate and that also etched away the ferromagnetic electrodes. Therefore, a polymer-based scheme has been developed and the flake can be suspended over long distances (Fig. 6B1). Moreover, researchers have discussed the reasons for the low spin relaxation time in detail [145]. Later, Drogeler et al. presented a new suspending way based on electrodes (Fig. 6B2). They firstly prepared the Co/MgO electrodes onto Si/SiO_2_ and then mechanically transferred a graphene/hBN heterostructure onto the pre-patterned electrodes. Furthermore, they explored room-temperature spin transport with different layers of graphene [146]. And by these means, even at a short transport channel of 2–3.5 μm, they achieved a spin relaxation time of 12.6 ns and a spin diffusion length of 30.5 μm [147], which are the maximum values of pristine graphene so far. It was also evidenced that the spin dephasing, caused by the solvent, was almost as important as the contact-induced dephasing. A recent suspending device with CVD monolayer graphene achieved a relaxation time of only 1.75 ns at room temperature [148]. Presumably, the discrepancy originated from the CVD graphene and the exfoliated graphene. On the other hand, utilizing highly polarized LSMO electrons, Yan et al. reported a spin valve with a few-layer graphene flake bridging electrodes that had a long spin diffusion length at low temperature [149].

Also, long-distance spin transport can be realized through a graphene quantum Hall antiferromagnet. Stepanov et al. reported a large non-local electrical signal across a 5-μm-long channel, where the utility of graphene in the quantum Hall regime was demonstrated [150]. In comparison with graphene, black phosphorus (BP) is a relatively new member of 2D materials with a sizeable direct band gap [2] (overcoming the lack of band gap in graphene) and considerable room-temperature mobilities (1000 cm^2^ V^−1^ s^−1^) [151] making it a promising transport material [152]. Moreover, phosphorus is a light element with weak SOC [153], indicating a long spin transport distance in theory. Avsar et al. fabricated a non-local spin valve with an hBN/BP/hBN structure, as shown in Fig. 7e, and obtained a spin relaxation time up to 4 ns and a spin diffusion length exceeding 6 μm at 100 K [154]. Besides, they established the basic spin properties of BP, to demonstrate that spin injection, transport, procession, and detection could be achieved in BP at room temperature.

In recent years, silicene has been attracting growing attention [155]. The different edges of silicene nanoribbons lead to rich possibilities of magnetic states, which bring in new opportunities for silicene to become spin transport channels [156]. In theory, the carrier mobility of silicene is up to 10,000 cm^2^ V^−1^ s^−1^ at room temperature [157]. Moreover, there are a large number of theoretical predictions proving that spin polarization and spin transport can be accomplished in silicene nanoribbons [155, 158, 159] and spin FET based on silicene nanoribbons was also proposed [158]. In the experiment, it has been reported that spin polarization was achieved in highly doped silicon at room temperature and the spin relaxation time was ~ 270 ps [107]. On the other hand, Li Tao et al. used a special growth–transfer–fabrication process (Fig. 7f) to implement FET at room temperature. Particularly, this approach addresses a major challenge for material preservation during transfer and device fabrication and is applicable to other air-sensitive 2D materials such as germanene and phosphorene [160]. In brief, these research outcomes encourage us to consider silicene as a promising candidate for efficient spin devices.

Apart from the traditional electronic transport channels mentioned above, a report suggested that 2D semiconductors can also act as a spin transport channel. Although the semiconductor channel remains challenging, Liang et al. have shown the evidence of electrical spin injection and detection in the conduction band of a multilayer MoS_2_, in which the spin diffusion length reached ~ 235 nm [161].

## Spin Manipulation in 2D Materials

Flexible control of the electron spin in materials is crucial to realize the practical application of spin devices [162]. Much of the effort has gone into exploiting effective manipulating schemes. Fortunately, 2D materials supply a wealth of means for spin manipulation, including magnetic engineering and proximity effect.

### Magnetic Control Engineering in 2D Materials

Starting from macroscopic magnetics is the most intuitive and direct way to manipulate the electron spin. Nevertheless, the practical application of this approach is not attractive. The magnetism of 2D materials is affected by many factors, providing abundant means for spin manipulation.

**Fig. 8:**
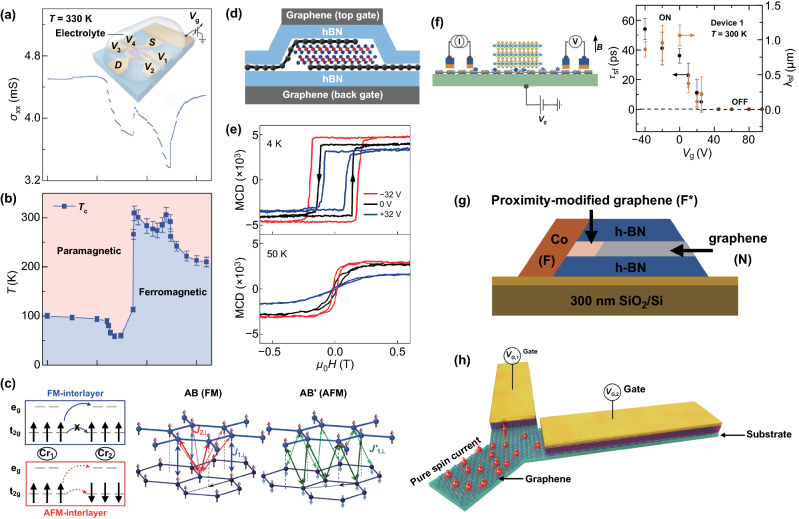
**a, b** Ferromagnetism in an atomically thin Fe_3_GeTe_2_ (FGT) flake modulated by an ionic gate. **a** Conductance as a function of the gate voltage Vg measured in a trilayer FGT device at room temperature. The inset shows a schematic of the FGT device structure. **b** Phase diagram of the trilayer FGT sample as the gate voltage and temperature are varied [99]. **c** Schematic of the orbital-dependent interlayer super-super-exchange interactions in CrI_3_ [167]. **d**, **e** 2D CrI_3_ field-effect devices. **d** A schematic side view of a dual-gate bilayer CrI_3_ field-effect device. **e** Magnetic circular dichroism versus magnetic field at three representative doping levels at 4 k (top panel) and 50 K (bottom panel) [163]. **f** Gate-controlled spin valve signal at room temperature [135]. **g** Schematic of the magnetic proximity effect for encapsulated graphene with a transparent 1D edge contact to a ferromagnetic electrode [173]. **h** A Y-shaped graphene spin current demultiplexer with gate voltages $V_{{{\mathrm{G}},i}}$ for voltage control of pure spin currents [175]. Figures reproduced with permission from Refs. [99, 135, 163, 167, 173, 175]

Firstly, electric control, through either an electric field or electrostatic doping, can change the electron population, orbit occupation, etc., leading to the modification of magnetic properties [14]. The 2D materials have an ultra-thin thickness, therefore, the external electric field can easily penetrate and change the magnetism of the 2D materials. For example, as shown in Fig. 8a, b, via ionic liquid gating, the $T_{{\mathrm{C}}}$ of Fe_3_GeTe_2_ exceeded room temperature [99], which is beneficial to the conductive electron-mediated ferromagnetism. In addition, by electrostatic doping (Fig. 8d, e), the bilayer CrI_3_ can be completely converted from interlayer antiferromagnetism to ferromagnetism [163]. And in the same manner, the $T_{{\mathrm{C}}}$ of monolayer CrI_3_ can be significantly regulated. Furthermore, if the magnetoelectric multiferroics of the materials are utilized, the electrical regulation efficiency of the magnetism could be significantly improved due to the inherent coupling of magnetic and electric orders. Unfortunately, there is little relevant theoretical information available.

Additionally, stress is an effective method to control the properties of materials, which is able to change the lattice structure of materials. Since the magnetic properties are closely related to the structural parameters of the material, such as the magnetostrictions, the stress also has a good regulation effect on the magnetic properties of the material. Spin–lattice coupling has been experimentally observed or theoretically predicted in layered magnets such as Cr_2_Si_2_Te_6_ [164], Cr_2_Ge_2_Te_6_ [165], and Fe_3_GeTe_2_ [166].

What’s more, the magnetic properties of magnetic 2D materials also strongly depend on the magnetic coupling between layers, implying that interlayer vdW interactions can be employed for magnetic control. For example, although the bulk CrI_3_ exhibits ferromagnetism, the interlayer magnetic interaction of the bilayer CrI_3_ exhibits antiferromagnetism. And the theory shows that the stacking between CrI_3_ monolayers determines the nature of interlayer magnetic coupling [167] (refer to Fig. 8c).

### Heterostructure in 2D Materials

As described earlier, the extremely weak SOC of graphene makes it the ideal spin transport channel. However, such weak SOC leads to the inability of electron spins in graphene, which limits its application in spin devices. As a result, enhancing the SOC of graphene has become a hot research direction, and many methods for improving the SOC of graphene have been proposed. For example, the covalently bonded hydrogen atoms can greatly enhance the spin–orbit interaction of the graphene [168]. But introducing adatoms will influence the transport of the spin electrons. Instead, the 2D material heterostructure, utilizing the proximity effect, is an ideal solution.

On the contrary, TMDCs have a strong SOC, and the heterostructure with graphene can significantly improve the SOC of graphene. For instance, in graphene with a WS_2_ substrate, there is a clear low-temperature weak antilocalization effect, which provides direct evidence of the enhanced SOC [8]. In the same way, TIs/graphene vdW heterostructure can lead to a strong proximity-induced SOC in graphene [169, 170]. Although an increasing number of methods have been adopted to enhance the SOC of graphene, there is still a lack of good schemes on how to manipulate spin transport. Recently, Yan et al. have successfully realized the manipulation of spin current in graphene by constructing a MoS_2_ on the graphene channel and they further explained its mechanism with the spin absorption theory [171]. Soon after, Dankert and Dash achieved manipulation at room temperature (Fig. 8f) that verified the spin absorption theory experimentally [135]. Nonetheless, true sense of utilizing SOC for precise spin manipulation as Datta and Das mentioned in 1990 [172] is still not realized.

In addition, the heterostructure of the spin channel and the ferromagnetic material can manipulate the spin by the exchange interaction between electrons. The exchange proximity interaction experienced by graphene in proximity to a ferromagnetic material acts as an effective Zeeman field for electrons in graphene that induces a spin precession around the magnetization axis of the ferromagnetic materials. Also, the magnetic properties of ferromagnetic materials can be controlled by electrical methods, making their practical applications possible [11]. Besides, utilizing a magnetic proximity effect of 1D ferromagnetic contact, Xu et al. demonstrated the gate-tunable spin transport. An electrostatic back gate can tune the Fermi level of graphene to probe different energy levels of the spin-polarized density of states of the 1D ferromagnetic contact (Fig. 8g). In contrast to conventional spin valves, they provided an alternative path to realize spin manipulation in graphene [173]. Recently, with first-principles calculation, Zollner has investigated the electronic band structure and the proximity exchange effect in bilayer graphene on a family of ferromagnetic multilayers of Cr_2_X_2_Te_6_ (X = Ge, Si, and Sn). They suggested that applying a vertical electric field reverses the exchange, allowing effective turning ON and OFF proximity magnetism in bilayer graphene [174].

Fascinatingly, different from the conventional manipulation methods, Lin et al. presented a mechanism of gate-driven pure current in graphene. Such a mechanism relies on electrical gating of carrier density-dependent conductivity and spin diffusion length in graphene and can realize the manipulation of spin current in a graphene spin current demultiplexer with a Y-shaped graphene channel [175], as shown in Fig. 8h.

## Application

2D materials provide a super-excellent platform for spintronic research. Besides, 2D materials have brought new prospects to the practical application of spintronics and are expected to make breakthroughs in low-power storage, computing, and communication.

First of all, 2D materials are broadly applied in magnetic tunnel junction (MTJ). The MTJ is a crucial component in spin devices and vital for implementing logic operations. And it is generally composed of two ferromagnetic layers separated by a tunnel barrier. The switch is realized by controlling the magnetization direction of the ferromagnetic layer to be parallel or antiparallel. Particularly, the uniform interface of the all-2D material MTJ can facilitate the all-area tunneling. Furthermore, based on the different properties of 2D materials, both the traditional MTJ and the MTJ with unique properties can be realized.

**Fig. 9:**
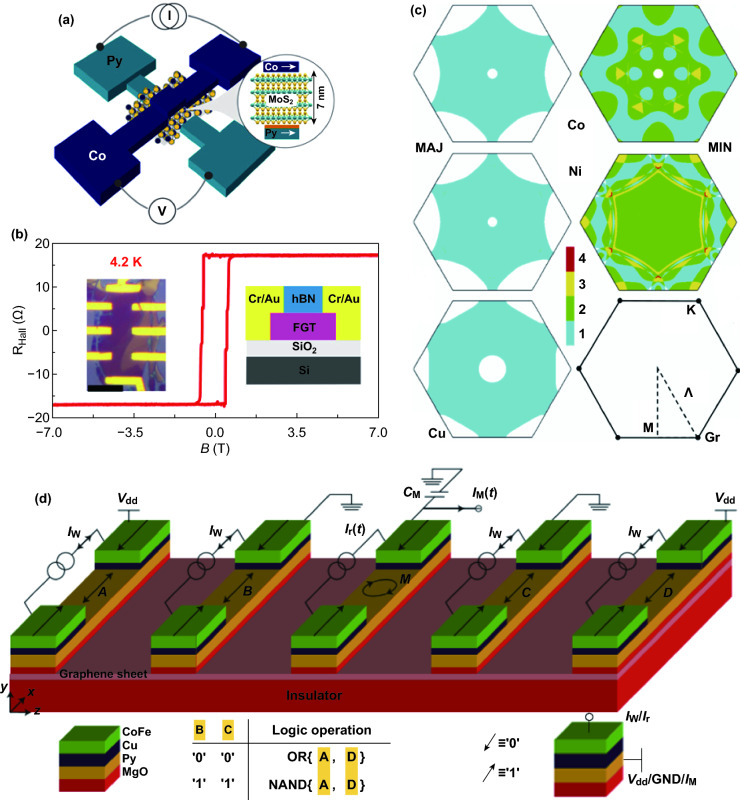
**a** Schematic representation with a multilayer MoS_2_ ferromagnetic contacts and a MoS_2_ spacer [182]. **b** Hall resistance of the tunneling spin valves based on Fe_3_GeTe_2_/hBN/Fe_3_GeTe_2_ heterostructure. The left and right insets are, respectively, an optical microscope image of the device and a schematic of the device cross section [183]. **c** Schematic of spin filtering effect in graphene. Fcc Fermi surface projections for Co (Ni, Cu) majority and minority spins. For graphene, surfaces of constant energy are centered on the k point [184]. **d** Schematic of graphene-based magnetologic consisting of a graphene sheet contacted by five ferromagnetic electrons. *A*/*D* is the input terminal. *M* is the output terminal. *B*/*C* is the controller (control logic gate type). *I*_w_ controls the magnetization direction of the electrode. *I*_r_ is used to disturb the magnetization direction of *M*. *I*_M_ is the transient response output current. V_dd_ is the steady-state input voltage, forming a loop with the ground of *B* (or *M*) and *C* (or *M*). Defining the magnetization direction “↑” as “1,” “↓” as “0” [15]. Figures reproduced with permission from Refs. [15, 182–184]

Conventionally, the tunnel barrier of MTJ is a metal oxide, such as Al_2_O_3_, MgO, and TiO_2_. However, it is difficult to avoid uneven metal oxide film during the growth process, which would result in low magnetoresistance. On the contrary, 2D materials have an atomically flat surface. Hence, as a tunnel barrier, the 2D insulator allows the spin flow to pass through uniformly, thereby achieving greater efficiency. For example, using CVD-hBN as a tunnel barrier, a magnetoresistance of 6% (${\text{MR}} = \left( {R_{{{\mathrm{AP}}}} - R_{{\mathrm{P}}} } \right)/R_{{\mathrm{P}}}$) was achieved at low temperature [176]. And recently Piquemal-Banci et al. have fabricated two illustrative systems (Co/CVD-hBN/Co and Co/CVD-hBN/Fe) and obtained magnetoresistance as high as 12% for Co/hBN/Co and 50% for Co/hBN/Fe. Furthermore, they analyzed these large values in light of spin filtering at hybrid chemisorbed/physisorbed hBN, in support of ab initio calculations [177]. Also, 2D semiconductors can act as a tunnel barrier, such as MoS_2_ [178–180] and WS_2_ [181]. Dankert et al. reported spin-polarized tunneling through a multilayer CVD MoS_2_ at room temperature (Fig. 9a) and observed a tunnel magnetoresistance of 0.5% [182]. In addition, by employing the heterostructure of a 2D ferromagnetic electrode of Fe_3_GeTe_2_ and hBN (Fig. 9b), the magnetoresistance could reach 160% at low temperature [183]. This structure makes the use of the advantages of the all-2D material MTJ, where the interface can achieve the all-area tunneling. Otherwise, according to the exponential function relationship between the tunneling current and the barrier thickness, the tunneling current tends to pass through the thinner area, resulting in a lower magnetoresistance [14]. Besides, 2D magnetic insulators have been proved to be effective to achieve large magnetoresistance. For example, with CrI_3_ as a tunnel barrier, a million percent magnetoresistance can be measured under low temperature in a strong magnetic field [89].

On the other hand, the perfect spin filtering effect of graphene can be well applied in MTJ. Due to the special lattice matching between graphene and some ferromagnetic electrodes (Co, Ni, etc.), only a minority of spin states can pass through graphene, and the majority of the spin states are filtered out [184, 185], as shown in Fig. 9c. In this way, a magnetoresistance of 12% at low temperatures is attained and magnetoresistance of 5% is still present at room temperature [186]. Moreover, a heterostructure of graphene/metal oxide film, as a tunneling barrier, can give rise to a large magnetoresistance. It has been reported that the atomic layer deposition technique could deposit Al_2_O_3_ film on CVD graphene, where the lattice structure of graphene was protected from damage, and the magnetoresistance at low temperature reached 31% [50]. Notably, most of the above researches could only get high magnetic resistance at low temperatures, and the switching states were regulated by external magnetic fields. Hence, there are still challenges limit the impending practical applications, but MTJ based on 2D materials has broad application prospects in spintronic devices.

Apart from MTJ, other conventional spintronic devices also rely on the regulation of external magnetic fields. Noticeably, the ideal device is able to achieve good electrical control with a small volume of critical current. The spin torque device based on the 2D materials can well meet this requirement, in which the interaction of spin-polarized electrons switches the magnetic states. Furthermore, by means of 2D materials, the device can be thinned toward atomic scale without causing the spin current dissipation in the body like in the bulk material [14]. In addition, 2D materials, such as MoS_2_ [187], can greatly enhance the perpendicular magnetic anisotropy of the ferromagnetic layer that is significant to overcome the thermal fluctuation.

The spin-transfer torque magnetic random access memory (STT-MRAM) [188], which has the advantages of non-volatility and fast storage speed, is a momentous application area for the spin torque device. STT-MRAM switches the logic state via changing the magnetization direction of the free layer with different spin polarization currents. However, traditional STT-MRAM cannot achieve the long-term goal of a smaller volume and write current. Therefore, achieving atomic thickness and a smaller switching critical current is essential for the potential application of the 2D materials for STT-MRAM.

Also, the unique advantages of 2D materials in electron spin transport, such as long enough spin relaxation time and spin diffusion length at room temperature, can be well applied. In addition to the previously mentioned graphene/TMDCs heterostructure field-effect transistors, various graphene-based FETs have been proposed theoretically and graphene-based spin logic devices have also been proposed, especially the graphene-based magnetologic gate [15, 122]. The structure of the magnetologic gate is shown in Fig. 9d, in which the output M is given by $\left\{ {\left( {A\;{\text{ XOR}}\;B} \right)\;{\text{OR}}\;\left( {C\;{\text{XOR}}\;D} \right)} \right\}$ (${\text{XOR}}$ and ${\text{OR}}$ present the logic gate operation). The "or gate" can be obtained by setting the states of *B* and *C* to "0" (magnetization direction "↓"). When the input of the *A* terminal is "1" (magnetization direction "↑"), the polarized direction of the spin current ("↑") injected into the graphene channel is opposite to that of the *B* terminal ("↓"). Due to the high resistance, the spin current ("↑") finally reaches the *M* terminal (non-polarized). Similarly, when the *D* input is "1" ("↑"), the spin flow ("↑") finally reaches the *M* end. Therefore, the output current at the *M* terminal is “0” only when the inputs of *A* and *D* are both "0," according to "or gate." On the contrary, when the states of *B* and *C* are set to "1" ("↑"), the "NAND gate" can be realized. Particularly, the magnetization direction of the electrode is managed by *I*_w_/*I*_r_ via spin torque.

The emergence of 2D materials has brought innovative platforms to spintronic devices, and various novel and interesting application ideas have been proposed. However, there are many restrictions to be overcome to realize the practical application of 2D material-based spintronic devices, including breakthroughs in room temperature, efficient and reliable spin manipulation, and mature processing techniques.

## Conclusion and Outlook

2D materials offer numerous prospects for spintronic development, making 2D material spintronics a fledgling field with infinite vitality. Based on several fundamental issues of spintronics, this review discusses the recent progress, future opportunities and challenges of spintronics in 2D materials.

First of all, the crucial conundrum is how to complete all-electric spin devices, which is aspirational for spintronic applications. From the spin injection point of view, an adequately enormous spin polarization has been obtained by the tunneling method, especially through hBN, or the optical method, with the assistance of the magnetic field for switching the FM electrodes or the incident light. In contrast, the SOC effect is capable of generating pure spin current via charge spin transition without the FM electrode. Nonetheless, the injection efficiency of this scheme is extremely low for spin devices at present. Additionally, the SOC effect is a central approach to manipulate spin for all-electric gate-tunable spin devices, which has not been accomplished yet. Recently, it has been demonstrated that the surfaces of 2D materials and the interfaces of heterostructures are crucial to the SOC effect. Therefore, a great effort should be directed to improve the surface and interface and further explore the more underlying characters of spintronics based on 2D materials.

On the other hand, spin relaxation is an imperative topic of 2D material spintronics, which is the key to optimize spin transport and dispose of the incompatibility between manipulation and transport. The current devices perform far below the theoretical value owing to the extrinsic factors that require better structure and fabrication process of the device, including hBN encapsulation, the transfer technique, and direct preparation of heterostructures by CVD. Although an hBN substrate is not able to upgrade the spin relaxation time $\tau$ directly, it can effectively enhance the mobility and the spin diffusion length $\lambda$ ($\lambda = \sqrt {\tau D}$). Also, annealing, which can get rid of the solvent residue, is a weighty technique to improve the performance of the device. In addition, there is still no consensus on the mechanism of spin relaxation in graphene. Therefore, the microcosmic picture of relaxation needs to be further explored, whereby the conflict between enhanced SOC and the long enough relaxation time could be managed well.

Notably, except for graphene, other 2D materials, such as BP, silicene, TMDCs, and their heterostructures, are all of the equal significance for transport channel study and application. Also, 2D magnetic materials with $T_{{\mathrm{C}}}$ exceeding room temperature are long-term goals for spin injection and manipulation at ambient conditions. Accordingly, there has been much effort going for achieving the high-temperature robust 2D magnetism and long-range ferromagnetic order. Meanwhile, there are various theoretically predicted materials to be explored. In light of existing results, it has been suggested that strengthening the exchange interaction and uniaxial magnetic anisotropy is the rule of thumb. As well, 2D TIs, which own strong SOC, are expected to play a significant role in spin devices. Beyond all doubts, the discovery of novel 2D materials can greatly enrich the spin effects and the ideas on device design.

**Fig. 10:**
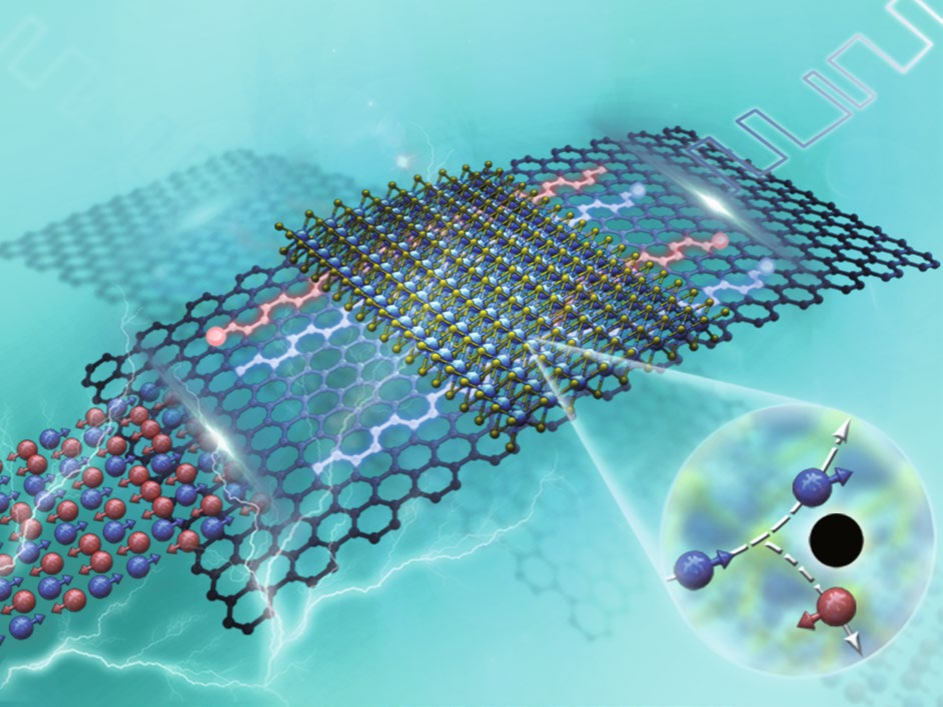
Schematic of the spintronics based on 2D materials. Electrons can be injected into 2D materials to generate spin current (the colors present the different spin) by electrical or optical injection, in which the electrons with different spins diffuse in the opposite direction. The spin could be manipulated by the interface effect of the 2D heterostructure to produce logic signals. (Color figure online)

Overall, 2D materials furnish a perfect platform for spintronics. Furthermore, the related heterostructures are a stepping stone for the research of 2D material spintronics, as shown in Fig. 10. Through decades of research, the achievements are quite attractive, but there are several snags that deserve attention. In short, the combination of 2D materials and spintronics presents an incredibly broad prospect and imperative value, not only for elementary theoretical research, but also for the advance of novel electronic devices.

## References

[1] Novoselov KS, Geim AK, Morozov SV, Jiang D, Zhang Y, Dubonos SV, Grigorieva IV, Firsov AA (2004). Electric field effect in atomically thin carbon films. Science.

[2] Liu H, Neal AT, Zhu Z, Luo Z, Xu XF, Tomanek D, Ye PD (2014). Phosphorene: an unexplored 2d semiconductor with a high hole mobility. ACS Nano.

[3] Wang QH, Kalantar-Zadeh K, Kis A, Coleman JN, Strano MS (2012). Electronics and optoelectronics of two-dimensional transition metal dichalcogenides. Nat. Nanotechnol..

[4] Guzman-Verri GG, Voon LCLY (2007). Electronic structure of silicon-based nanostructures. Phys. Rev. B.

[5] Geim AK, Grigorieva IV (2013). Van der waals heterostructures. Nature.

[6] Novoselov KS, Mishchenko A, Carvalho A, Neto AHC (2016). 2d materials and van der waals heterostructures. Science.

[7] Garcia JH, Vila M, Cummings AW, Roche S (2018). Spin transport in graphene/transition metal dichalcogenide heterostructures. Chem. Soc. Rev..

[8] Wang Z, Ki DK, Chen H, Berger H, MacDonald AH, Morpurgo AF (2015). Strong interface-induced spin–orbit interaction in graphene on WS_2_. Nat Commun..

[9] Garcia JH, Cummings AW, Roche S (2017). Spin hall effect and weak antilocalization in graphene/transition metal dichalcogenide heterostructures. Nano Lett..

[10] Han W (2016). Perspectives for spintronics in 2d materials. Appl. Mater..

[11] Feng YP, Shen L, Yang M, Wang AZ, Zeng MG, Wu QY, Chintalapati S, Chang CR (2017). Prospects of spintronics based on 2d materials. Wires Comput. Mol. Sci..

[12] Iqbal MZ, Qureshi NA, Hussain G (2018). Recent advancements in 2d-materials interface based magnetic junctions for spintronics. J. Magn. Magn. Mater..

[13] Tan CL, Cao XH, Wu XJ, He QY, Yang J (2017). Recent advances in ultrathin two-dimensional nanomaterials. Chem. Rev..

[14] Gong C, Zhang X (2019). Two-dimensional magnetic crystals and emergent heterostructure devices. Science.

[15] Han W, Kawakami RK, Gmitra M, Fabian J (2014). Graphene spintronics. Nat. Nanotechnol..

[16] Yazyev OV, Helm L (2007). Defect-induced magnetism in graphene. Phys. Rev. B.

[17] McCreary KM, Swartz AG, Han W, Fabian J, Kawakami RK (2012). Magnetic moment formation in graphene detected by scattering of pure spin currents. Phys. Rev. Lett..

[18] Giesbers AJM, Uhlirova K, Konecny M, Peters EC, Burghard M, Aarts J, Flipse CFJ (2013). Interface-induced room-temperature ferromagnetism in hydrogenated epitaxial graphene. Phys. Rev. Lett..

[19] Boukhvalov DW, Katsnelson MI, Lichtenstein AI (2008). Hydrogen on graphene: Electronic structure, total energy, structural distortions and magnetism from first-principles calculations. Phys. Rev. B.

[20] Nair RR, Sepioni M, Tsai IL, Lehtinen O, Keinonen J, Krasheninnikov AV, Thomson T, Geim AK, Grigorieva IV (2012). Spin-half paramagnetism in graphene induced by point defects. Nat. Phys..

[21] Cervenka J, Katsnelson MI, Flipse CFJ (2009). Room-temperature ferromagnetism in graphite driven by two-dimensional networks of point defects. Nat. Phys..

[22] Yazyev OV, Katsnelson MI (2008). Magnetic correlations at graphene edges: basis for novel spintronics devices. Phys. Rev. Lett..

[23] Jung J, Pereg-Barnea T, MacDonald AH (2009). Theory of interedge superexchange in zigzag edge magnetism. Phys. Rev. Lett..

[24] Son YW, Cohen ML, Louie SG (2006). Half-metallic graphene nanoribbons. Nature.

[25] Elias DC, Nair RR, Mohiuddin TMG, Morozov SV, Blake P (2009). Control of graphene's properties by reversible hydrogenation: evidence for graphane. Science.

[26] Pumera M, Wong CHA (2013). Graphane and hydrogenated graphene. Chem. Soc. Rev..

[27] Lazar P, Karlicky F, Jurečka P, Kocman MS, Otyepková E, Šafářová KR, Otyepka M (2013). Adsorption of small organic molecules on grapheme. J. Am. Chem. Soc..

[28] Rubio-Pereda P, Takeuchi N (2016). Van der waals molecular interactions in the organic functionalization of graphane, silicane, and germanane with alkene and alkyne molecules: a DFT-D2 study. J. Mol. Model..

[29] Li WF, Zhao MW, He T, Song C, Lin XH, Liu XD, Xia YY, Mei LM (2010). Concentration dependent magnetism induced by hydrogen adsorption on graphene and single walled carbon nanotubes. J. Magn. Magn. Mater..

[30] Li WF, Zhao MW, Xia YY, Zhang RQ, Mu YG (2009). Covalent-adsorption induced magnetism in graphene. J. Mater. Chem..

[31] Wehling TO, Katsnelson MI, Lichtenstein AI (2009). Adsorbates on graphene: Impurity states and electron scattering. Chem. Phys. Lett..

[32] Casolo S, Lovvik OM, Martinazzo R, Tantardini GF (2009). Understanding adsorption of hydrogen atoms on graphene. J. Chem. Phys..

[33] Zheng YP, Wan XG, Tang NJ, Feng Q, Liu FC, Du YW (2015). Magnetic properties of double-side partially fluorinated graphene from first principles calculations. Carbon.

[34] Yazyev OV (2008). Magnetism in disordered graphene and irradiated graphite. Phys. Rev. Lett..

[35] Dev P, Reinecke TL (2014). Substrate effects: disappearance of adsorbate-induced magnetism in graphene. Phys. Rev. B.

[36] Sepioni M, Nair RR, Tsai IL, Geim AK, Grigorieva IV (2012). Revealing common artifacts due to ferromagnetic inclusions in highly oriented pyrolytic graphite. EPL-Europhys. Lett..

[37] Sepioni M, Nair RR, Rablen S, Narayanan J, Tuna F, Winpenny R, Geim AK, Grigorieva IV (2010). Limits on intrinsic magnetism in graphene. Phys. Rev. Lett..

[38] Stauber T, Peres NMR, Guinea F, Castro AH (2007). Fermi liquid theory of a fermi ring. Phys. Rev. B.

[39] Castro EV, Peres NMR, Stauber T, Silva NAP (2008). Low-density ferromagnetism in biased bilayer graphene. Phys. Rev. Lett..

[40] Cao T, Li ZL, Louie SG (2015). Tunable magnetism and half-metallicity in hole-doped monolayer gase. Phys. Rev. Lett..

[41] Wang ZY, Tang C, Sachs R, Barlas Y, Shi J (2015). Proximity-induced ferromagnetism in graphene revealed by the anomalous hall effect. Phys. Rev. Lett..

[42] Wei P, Lee S, Lemaitre F, Pinel L, Cutaia D (2016). Strong interfacial exchange field in the graphene/EuS heterostructure. Nat. Mater..

[43] Xu L, Yang M, Shen L, Zhou J, Zhu T, Feng YP (2018). Large valley splitting in monolayer WS_2_ by proximity coupling to an insulating antiferromagnetic substrate. Phys. Rev. B.

[44] Maassen J, Ji W, Guo H (2011). Graphene spintronics: the role of ferromagnetic electrodes. Nano Lett..

[45] Han W, Pi K, McCreary KM, Li Y, Wong JJI, Swartz AG, Kawakami RK (2010). Tunneling spin injection into single layer graphene. Phys. Rev. Lett..

[46] Han W, McCreary KM, Pi K, Wang WH, Li Y, Wen H, Chen JR, Kawakami RK (2012). Spin transport and relaxation in graphene. J. Magn. Magn. Mater..

[47] Schmidt G, Ferrand D, Molenkamp LW, Filip AT, van Wees BJ (2000). Fundamental obstacle for electrical spin injection from a ferromagnetic metal into a diffusive semiconductor. Phys. Rev. B.

[48] Rashba EI (2000). Theory of electrical spin injection: Tunnel contacts as a solution of the conductivity mismatch problem. Phys. Rev. B.

[49] Fert A, Jaffres H (2001). Conditions for efficient spin injection from a ferromagnetic metal into a semiconductor. Phys. Rev. B.

[50] Martin MB, Dlubak B, Weatherup RS, Yang H, Deranlot C (2014). Sub-nanometer atomic layer deposition for spintronics in magnetic tunnel junctions based on graphene spin-filtering membranes. ACS Nano.

[51] Han W, Pi K, Bao W, McCreary K, Li Y, Wang W, Lau C, Kawakami R (2009). Electrical detection of spin precession in single layer graphene spin valves with transparent contacts. Appl. Phys. Lett..

[52] Tombros N, Jozsa C, Popinciuc M, Jonkman HT, van Wees BJ (2007). Electronic spin transport and spin precession in single graphene layers at room temperature. Nature.

[53] Drögeler M, Volmer F, Wolter M, Terrés B, Watanabe K (2014). Nanosecond spin lifetimes in single- and few-layer graphene–hBN heterostructures at room temperature. Nano Lett..

[54] Volmer F, Drogeler M, Maynicke E, von den Driesch N, Boschen ML, Guntherodt G, Beschoten B (2013). Role of MgO barriers for spin and charge transport in Co/MgO/graphene nonlocal spin-valve devices. Phys. Rev. B.

[55] Popinciuc M, Jozsa C, Zomer PJ, Tombros N, Veligura A, Jonkman HT, van Wees BJ (2009). Electronic spin transport in graphene field-effect transistors. Phys. Rev. B.

[56] Jozsa C, Popinciuc M, Tombros N, Jonkman HT, van Wees BJ (2008). Electronic spin drift in graphene field-effect transistors. Phys. Rev. Lett..

[57] Dlubak B, Seneor P, Anane A, Barraud C, Deranlot C (2010). Are Al_2_O_3_ and MgO tunnel barriers suitable for spin injection in graphene?. Appl. Phys. Lett..

[58] Yamaguchi T, Masubuchi S, Iguchi K, Moriya R, Machida T (2012). Tunnel spin injection into graphene using Al_2_O_3_ barrier grown by atomic layer deposition on functionalized graphene surface. J. Magn. Magn. Mater..

[59] Jozsa C, Popinciuc M, Tombros N, Jonkman HT, van Wees BJ (2009). Controlling the efficiency of spin injection into graphene by carrier drift. Phys. Rev. B.

[60] Jozsa C, Maassen T, Popinciuc M, Zomer PJ, Veligura A, Jonkman HT, van Wees BJ (2009). Linear scaling between momentum and spin scattering in graphene. Phys. Rev. B.

[61] Wang WH, Pi K, Li Y, Chiang YF, Wei P, Shi J, Kawakami RK (2008). Magnetotransport properties of mesoscopic graphite spin valves. Phys. Rev. B.

[62] Volmer F, Drogeler M, Guntherodt G, Stampfer C, Beschoten B (2015). Spin and charge transport in graphene-based spin transport devices with Co/MgO spin injection and spin detection electrodes. Synth. Metals.

[63] Volmer F, Drogeler M, Pohlmann T, Guntherodt G, Stampfer C, Beschoten B (2015). Contact-induced charge contributions to non-local spin transport measurements in Co/MgO/graphene devices. 2D Mater..

[64] Cubukcu M, Martin M-B, Laczkowski P, Vergnaud C, Marty A (2015). Ferromagnetic tunnel contacts to graphene: contact resistance and spin signal. J. Appl. Phys..

[65] Dankert A, Kamalakar MV, Bergsten J, Dash SP (2014). Spin transport and precession in graphene measured by nonlocal and three-terminal methods. Appl. Phys. Lett..

[66] Liu YP, Idzuchi H, Fukuma Y, Rousseau O, Otani Y, Lew WS (2013). Spin injection properties in trilayer graphene lateral spin valves. Appl. Phys. Lett..

[67] Dlubak B, Martin MB, Deranlot C, Bouzehouane K, Fusil S (2012). Homogeneous pinhole free 1 nm Al_2_O_3_ tunnel barriers on graphene. Appl. Phys. Lett..

[68] Wu QY, Shen L, Bai ZQ, Zeng MG, Yang M, Huang ZG, Feng YP (2014). Efficient spin injection into graphene through a tunnel barrier: overcoming the spin-conductance mismatch. Phys. Rev. Appl..

[69] Xue J, Sanchez-Yamagishi J, Bulmash D, Jacquod P, Deshpande A (2011). Scanning tunnelling microscopy and spectroscopy of ultra-flat graphene on hexagonal boron nitride. Nat. Mater..

[70] Giovannetti G, Khomyakov PA, Brocks G, Kelly PJ, van den Brink J (2007). Substrate-induced band gap in graphene on hexagonal boron nitride: ab initio density functional calculations. Phys. Rev. B.

[71] Neumann C, Reichardt S, Venezuela P, Drogeler M, Banszerus L (2015). Raman spectroscopy as probe of nanometre-scale strain variations in graphene. Nat. Commun..

[72] Singh S, Katoch J, Xu JS, Tan C, Zhu TC, Amamou W, Hone J, Kawakami R (2016). Nanosecond spin relaxation times in single layer graphene spin valves with hexagonal boron nitride tunnel barriers. Appl. Phys. Lett..

[73] Yamaguchi T, Inoue Y, Masubuchi S, Morikawa S, Onuki M (2013). Electrical spin injection into graphene through monolayer hexagonal boron nitride. Appl. Phys. Express.

[74] Kamalakar MV, Dankert A, Bergsten J, Ive T, Dash SP (2014). Enhanced tunnel spin injection into graphene using chemical vapor deposited hexagonal boron nitride. Sci. Rep..

[75] Gurram M, Omar S, Zihlmann S, Makk P, Li QC, Zhang YF, Schonenberger C, van Wees BJ (2018). Spin transport in two-layer-CVD-hbn/graphene/hbn heterostructures. Phys. Rev. B.

[76] Fu W, Makk P, Maurand R, Bräuninger M, Schönenberger C (2014). Large-scale fabrication of BN tunnel barriers for graphene spintronics. J. Appl. Phys..

[77] Kamalakar MV, Dankert A, Bergsten J, Ive T, Dash SP (2014). Spintronics with graphene-hexagonal boron nitride van der waals heterostructures. Appl. Phys. Lett..

[78] Britnell L, Gorbachev RV, Jalil R, Belle BD, Schedin F (2012). Electron tunneling through ultrathin boron nitride crystalline barriers. Nano Lett..

[79] Leutenantsmeyer JC, Ingla-Aynes J, Gurram M, van Wees BJ (2018). Efficient spin injection into graphene through trilayer hbn tunnel barriers. J. Appl. Phys..

[80] Zomer P, Guimarães M, Brant J, Tombros N, Van Wees B (2014). Fast pick up technique for high quality heterostructures of bilayer graphene and hexagonal boron nitride. Appl. Phys. Lett..

[81] Liu YP, Zhang SY, He J, Wang ZMM, Liu ZW (2019). Recent progress in the fabrication, properties, and devices of heterostructures based on 2d materials. Nano Micro Lett..

[82] Kamalakar MV, Dankert A, Kelly PJ, Dash SP (2016). Inversion of spin signal and spin filtering in ferromagnet vertical bar hexagonal boron nitride-graphene van der waals heterostructures. Sci. Rep..

[83] Gurram M, Omar S, van Wees BJ (2017). Bias induced up to 100% spin-injection and detection polarizations in ferromagnet/bilayer-hBN/graphene/hBN heterostructures. Nat. Commun..

[84] Friedman AL, van't Erve OMJ, Li CH, Robinson JT, Jonker BT (2014). Homoepitaxial tunnel barriers with functionalized graphene-on-graphene for charge and spin transport. Nat. Commun..

[85] Vaklinova K, Hoyer A, Burghard M, Kern K (2016). Current-induced spin polarization in topological insulator-graphene heterostructures. Nano Lett..

[86] Song TC, Cai XH, Tu MWY, Zhang XO, Huang BV (2018). Giant tunneling magnetoresistance in spin-filter van der waals heterostructures. Science.

[87] Klein DR, MacNeill D, Lado JL, Soriano D, Navarro-Moratalla E (2018). Probing magnetism in 2d van der waals crystalline insulators via electron tunneling. Science.

[88] Wang Z, Gutierrez-Lezama I, Ubrig N, Kroner M, Gibertini M (2018). Very large tunneling magnetoresistance in layered magnetic semiconductor CrI_3_. Nat Commun..

[89] Kim HH, Yang BW, Patel T, Sfigakis F, Li CH, Tian SJ, Lei HC, Tsen AW (2018). One million percent tunnel magnetoresistance in a magnetic van der waals heterostructure. Nano Lett..

[90] Ghazaryan D, Greenaway MT, Wang Z, Guarochico-Moreira VH, Vera-Marun IJ (2018). Magnon-assisted tunnelling in van der waals heterostructures based on CrBr_3_. Nat. Electron..

[91] Yamaguchi T, Moriya R, Oki S, Yamada S, Masubuchi S, Hamaya K, Machida T (2016). Spin injection into multilayer graphene from highly spin-polarized Co2FeSi heusler alloy. Appl. Phys. Express.

[92] Aseev PP, Artemenko SN (2015). Spin injection from topological insulator into metal leads. Physica B.

[93] Sun QL, Kioussis N (2018). Prediction of manganese trihalides as two-dimensional dirac half-metals. Phys. Rev. B.

[94] Ashton M, Gluhovic D, Sinnott SB, Guo J, Stewart DA, Hennig RG (2017). Two-dimensional intrinsic half-metals with large spin gaps. Nano Lett..

[95] He JJ, Li S (2018). Two-dimensional janus transition-metal dichalcogenides with intrinsic ferromagnetism and half-metallicity. Comput. Mater. Sci..

[96] Gong SJ, Gong C, Sun YY, Tong WY, Duan CG, Chu JH, Zhang X (2018). Electrically induced 2D half-metallic antiferromagnets and spin field effect transistors. Proc. Natl. Acad. Sci. USA.

[97] Bonilla M, Kolekar S, Ma YJ, Diaz HC, Kalappattil V (2018). Strong room-temperature ferromagnetism in VSe_2_ monolayers on van der waals substrates. Nat. Nanotechnol..

[98] O'Hara DJ, Zhu TC, Trout AH, Ahmed AS, Luo YK (2018). Room temperature intrinsic ferromagnetism in epitaxial manganese selenide films in the monolayer limit. Nano Lett..

[99] Deng YJ, Yu YJ, Song YC, Zhang JZ, Wang NZ (2018). Gate-tunable room-temperature ferromagnetism in two-dimensional Fe_3_GeTe_2_. Nature.

[100] Inglot M, Dugaev VK, Sherman EY, Barnas J (2014). Optical spin injection in graphene with rashba spin–orbit interaction. Phys. Rev. B.

[101] Rioux J, Burkard G (2014). Photoinduced pure spin-current injection in graphene with rashba spin–orbit interaction. Phys. Rev. B.

[102] Liu Y, Gao Y, Zhang S, He J, Yu J, Liu Z (2019). Valleytronics in transition metal dichalcogenides materials. Nano Res..

[103] Luo YK, Xu JS, Zhu TC, Wu GZ, McCormick EJ (2017). Opto-valleytronic spin injection in monolayer MoS_2_/few-layer graphene hybrid spin valves. Nano Lett..

[104] Avsar A, Unuchek D, Liu JW, Sanchez OL, Watanabe K, Taniguch T, Ozyilmaz B, Kis A (2017). Optospintronics in graphene via proximity coupling. ACS Nano.

[105] Cheng L, Wang XB, Yang WF, Chai JW, Yang M (2019). Far out-of-equilibrium spin populations trigger giant spin injection into atomically thin MoS_2_. Nat. Phys..

[106] Ando K, Takahashi S, Ieda J, Kurebayashi H, Trypiniotis T, Barnes CHW, Maekawa S, Saitoh E (2011). Electrically tunable spin injector free from the impedance mismatch problem. Nat. Mater..

[107] Dash SP, Sharma S, Patel RS, de Jong MP, Jansen R (2009). Electrical creation of spin polarization in silicon at room temperature. Nature.

[108] Jonker BT, Kioseoglou G, Hanbicki AT, Li CH, Thompson PE (2007). Electrical spin-injection into silicon from a ferromagnetic metal/tunnel barrier contact. Nat. Phys..

[109] Battiato M, Held K (2016). Ultrafast and gigantic spin injection in semiconductors. Phys. Rev. Lett..

[110] Soumyanarayanan A, Reyren N, Fert A, Panagopoulos C (2016). Emergent phenomena induced by spin–orbit coupling at surfaces and interfaces. Nature.

[111] Edelstein VM (1990). Spin polarization of conduction electrons induced by electric current in two-dimensional asymmetric electron systems. Solid State Commun..

[112] Hoffmann A (2013). Spin hall effects in metals. IEEE Trans. Magn..

[113] Rojas-Sanchez JC, Oyarzun S, Fu Y, Marty A, Vergnaud C (2016). Spin to charge conversion at room temperature by spin pumping into a new type of topological insulator: alpha-Sn films. Phys. Rev. Lett..

[114] Xu Y, Yan BH, Zhang HJ, Wang J, Xu G, Tang PZ, Duan WH, Zhang SC (2013). Large-gap quantum spin hall insulators in tin films. Phys. Rev. Lett..

[115] Zhu FF, Chen WJ, Xu Y, Gao CL, Guan DD (2015). Epitaxial growth of two-dimensional stanene. Nat. Mater..

[116] Tang PZ, Chen PC, Cao WD, Huang HQ, Cahangirov S (2014). Stable two-dimensional dumbbell stanene: a quantum spin hall insulator. Phys. Rev. B.

[117] Liu CC, Feng WX, Yao YG (2011). Quantum spin hall effect in silicene and two-dimensional germanium. Phys. Rev. Lett..

[118] Song ZG, Liu CC, Yang JB, Han JZ, Ye M (2014). Quantum spin hall insulators and quantum valley hall insulators of Bix/Sbx (x = H, F, Cl and Br) monolayers with a record bulk band gap. NPG Asia Mater..

[119] Safeer CK, Ingla-Aynes J, Herling F, Garcia JH, Vila M (2019). Room-temperature spin hall effect in graphene/MoS_2_ van der waals heterostructures. Nano Lett..

[120] Yan WJ, Sagasta E, Ribeiro M, Niimi Y, Hueso LE, Casanova F (2017). Large room temperature spin-to-charge conversion signals in a few-layer graphene/Pt lateral heterostructure. Nat. Commun..

[121] Roche S, Valenzuela SO (2014). Graphene spintronics: puzzling controversies and challenges for spin manipulation. J. Phys. D Appl. Phys..

[122] Dery H, Wu H, Ciftcioglu B, Huang M, Song Y (2012). Nanospintronics based on magnetologic gates. IEEE Trans. Electron. Dev..

[123] Liu YP, Goolaup S, Murapaka C, Lew WS, Wong SK (2010). Effect of magnetic field on the electronic transport in trilayer graphene. ACS Nano.

[124] Liu YP, Liu X, Zhang YJ, Xia QL, He J (2017). Effect of magnetic field on electronic transport in a bilayer graphene nanomesh. Nanotechnology.

[125] Gurram M, Omar S, van Wees BJ (2018). Electrical spin injection, transport, and detection in graphene-hexagonal boron nitride van der waals heterostructures: progress and perspectives. 2D Mater..

[126] Sabio J, Seoanez C, Fratini S, Guinea F, Castro AH, Sols F (2008). Electrostatic interactions between graphene layers and their environment. Phys. Rev. B.

[127] Chen JH, Jang C, Adam S, Fuhrer MS, Williams ED, Ishigami M (2008). Charged-impurity scattering in graphene. Nat. Phys..

[128] Tuan DV, Ortmann F, Cummings AW, Soriano D, Roche S (2016). Spin dynamics and relaxation in graphene dictated by electron-hole puddles. Sci. Rep..

[129] Martin J, Akerman N, Ulbricht G, Lohmann T, Smet JH, Von Klitzing K, Yacoby A (2008). Observation of electron-hole puddles in graphene using a scanning single-electron transistor. Nat. Phys..

[130] Maassen T, van den Berg JJ, IJbema N, Fromm F, Seyller T, Yakimova R, van Wees BJ (2012). Long spin relaxation times in wafer scale epitaxial graphene on sic(0001). Nano Lett..

[131] Dlubak B, Martin MB, Deranlot C, Servet B, Xavier S (2012). Highly efficient spin transport in epitaxial graphene on sic. Nat. Phys..

[132] Leutenantsmeyer JC, Kaverzin AA, Wojtaszek M, van Wees BJ (2017). Proximity induced room temperature ferromagnetism in graphene probed with spin currents. J Mater..

[133] Singh S, Katoch J, Zhu TC, Meng KY, Liu TY (2017). Strong modulation of spin currents in bilayer graphene by static and fluctuating proximity exchange fields. Phys. Rev. Lett..

[134] Omar S, van Wees BJ (2017). Graphene-ws2 heterostructures for tunable spin injection and spin transport. Phys. Rev. B.

[135] Dankert A, Dash SP (2017). Electrical gate control of spin current in van der waals heterostructures at room temperature. Nat. Commun..

[136] Maassen T, van den Berg JJ, Huisman EH, Dijkstra H, Fromm F, Seyller T, van Wees BJ (2013). Localized states influence spin transport in epitaxial graphene. Phys. Rev. Lett..

[137] Zomer PJ, Guimaraes MHD, Tombros N, van Wees BJ (2012). Long-distance spin transport in high-mobility graphene on hexagonal boron nitride. Phys. Rev. B.

[138] Han W, Chen JR, Wang DQ, McCreary KM, Wen H, Swartz AG, Shi J, Kawakami RK (2012). Spin relaxation in single-layer graphene with tunable mobility. Nano Lett..

[139] Castro Neto AH, Guinea F (2009). Impurity-induced spin–orbit coupling in graphene. Phys. Rev. Lett..

[140] Guimaraes MHD, Zomer PJ, Ingla-Aynes J, Brant JC, Tombros N, van Wees BJ (2014). Controlling spin relaxation in hexagonal bn-encapsulated graphene with a transverse electric field. Phys. Rev. Lett..

[141] Gurram M, Omar S, Zihlmann S, Makk P, Schonenberger C, van Wees BJ (2016). Spin transport in fully hexagonal boron nitride encapsulated graphene. Phys. Rev. B.

[142] Serrano IG, Panda J, Denoel F, Vallin O, Phuyal D, Karis O, Kamalakar MV (2019). Two-dimensional flexible high diffusive spin circuits. Nano Lett..

[143] Bolotin KI, Sikes KJ, Jiang Z, Klima M, Fudenberg G, Hone J, Kim P, Stormer HL (2008). Ultrahigh electron mobility in suspended graphene. Solid State Commun..

[144] Du X, Skachko I, Barker A, Andrei EY (2008). Approaching ballistic transport in suspended graphene. Nat. Nanotechnol..

[145] Guimaraes MHD, Veligura A, Zomer PJ, Maassen T, Vera-Marun IJ, Tombros N, van Arees BJ (2012). Spin transport in high-quality suspended graphene devices. Nano Lett..

[146] Drogeler M, Volmer F, Wolter M, Terres B, Watanabe K (2014). Nanosecond spin lifetimes in single- and few-layer graphene-hbn heterostructures at room temperature. Nano Lett..

[147] Drogeler M, Franzen C, Volmer F, Pohlmann T, Banszerus L (2016). Spin lifetimes exceeding 12 ns in graphene nonlocal spin valve devices. Nano Lett..

[148] Drogeler M, Banszerus L, Volmer F, Taniguchi T, Watanabe K, Beschoten B, Stampfer C (2017). Dry-transferred cvd graphene for inverted spin valve devices. Appl. Phys. Lett..

[149] Yan W, Phillips L, Barbone M, Hämäläinen S, Lombardo A (2016). Long spin diffusion length in few-layer graphene flakes. Phys. Rev. Lett..

[150] Stepanov P, Che S, Shcherbakov D, Yang JW, Chen RY (2018). Long-distance spin transport through a graphene quantum hall antiferromagnet. Nat. Phys..

[151] Long G, Maryenko D, Shen JY, Xu SG, Hou JQ (2016). Achieving ultrahigh carrier mobility in two-dimensional hole gas of black phosphorus. Nano Lett..

[152] Xiang Y, Xia QL, Luo JH, Liu YP, Peng YD, Wang DW, Nie YZ, Guo GH (2018). Observation of ferromagnetism in black phosphorus nanosheets with high magnetization by liquid exfoliation. Solid State Commun..

[153] Popovic ZS, Kurdestany JM, Satpathy S (2015). Electronic structure and anisotropic rashba spin–orbit coupling in monolayer black phosphorus. Phys. Rev. B.

[154] Avsar A, Tan JY, Kurpas M, Gmitra M, Watanabe K, Taniguchi T, Fabian J, Ozyilmaz B (2017). Gate-tunable black phosphorus spin valve with nanosecond spin lifetimes. Nat. Phys..

[155] Xu N (2018). Spin-polarized transport in multiterminal silicene nanodevices. Phys. Lett. A.

[156] Zhao JJ, Liu HS, Yu ZM, Quhe RG, Zhou S (2016). Rise of silicene: a competitive 2D material. Prog. Mater. Sci..

[157] Shao ZG, Ye XS, Yang L, Wang CL (2013). First-principles calculation of intrinsic carrier mobility of silicene. J. Appl. Phys..

[158] Pournaghavi N, Esmaeilzadeh M, Abrishamifar A, Ahmadi S (2017). Extrinsic Rashba spin–orbit coupling effect on silicene spin polarized field effect transistors. J. Phys.-Condens. Matter.

[159] Shakouri K, Simchi H, Esmaeilzadeh M, Mazidabadi H, Peeters FM (2015). Tunable spin and charge transport in silicene nanoribbons. Phys. Rev. B.

[160] Tao L, Cinquanta E, Chiappe D, Grazianetti C, Fanciulli M, Dubey M, Molle A, Akinwande D (2015). Silicene field-effect transistors operating at room temperature. Nat. Nanotechnol..

[161] Liang SH, Yang HW, Renucci P, Tao BS, Laczkowski P (2017). Electrical spin injection and detection in molybdenum disulfide multilayer channel. Nat. Commun..

[162] Roche S, Åkerman J, Beschoten B, Charlier J-C, Chshiev M (2015). Graphene spintronics: the European flagship perspective. 2D Mater..

[163] Jiang SW, Li LZ, Wang ZF, Mak KF, Shan J (2018). Controlling magnetism in 2D CrI_3_ by electrostatic doping. Nat. Nanotechnol..

[164] Casto LD, Clune AJ, Yokosuk MO, Musfeldt JL, Williams TJ (2015). Strong spin-lattice coupling in CrSiTe_3_. APL Mater..

[165] Tian Y, Gray MJ, Ji HW, Cava RJ, Burch KS (2016). Magneto-elastic coupling in a potential ferromagnetic 2d atomic crystal. 2D Mater..

[166] Zhuang HLL, Kent PRC, Hennig RG (2016). Strong anisotropy and magnetostriction in the two-dimensional stoner ferromagnet Fe_3_GeTe_2_. Phys. Rev. B.

[167] Sivadas N, Okamoto S, Xu XD, Fennie CJ, Xiao D (2018). Stacking-dependent magnetism in bilayer CrI_3_. Nano Lett..

[168] Balakrishnan J, Koon GKW, Jaiswal M, Neto AC, Özyilmaz B (2013). Colossal enhancement of spin–orbit coupling in weakly hydrogenated graphene. Nat. Phys..

[169] Khokhriakov D, Cummings AW, Song K, Vila M, Karpiak B, Dankert A, Roche S, Dash SP (2018). Tailoring emergent spin phenomena in dirac material heterostructures. Sci. Adv..

[170] Song K, Soriano D, Cummings AW, Robles R, Ordejón P, Roche S (2018). Spin proximity effects in graphene/topological insulator heterostructures. Nano Lett..

[171] Yan WJ, Txoperena O, Llopis R, Dery H, Hueso LE, Casanova F (2016). A two-dimensional spin field-effect switch. Nat. Commun..

[172] Datta S, Das B (1990). Electronic analog of the electro-optic modulator. Appl. Phys. Lett..

[173] Xu JS, Singh S, Katoch J, Wu GZ, Zhu TC, Zutic I, Kawakami RK (2018). Spin inversion in graphene spin valves by gate-tunable magnetic proximity effect at one-dimensional contacts. Nat. Commun..

[174] Zollner K, Gmitra M, Fabian J (2018). Electrically tunable exchange splitting in bilayer graphene on monolayer Cr_2_X_2_Te_6_ with X = Ge, Si, and Sn. New J. Phys..

[175] Lin XY, Su L, Si ZZ, Zhang YG, Bournel A (2017). Gate-driven pure spin current in graphene. Phys. Rev. Appl..

[176] Piquemal-Banci M, Galceran R, Caneva S, Martin MB, Weatherup RS (2016). Magnetic tunnel junctions with monolayer hexagonal boron nitride tunnel barriers. Appl. Phys. Lett..

[177] Piquemal-Banci M, Galceran R, Godel F, Caneva S, Martin MB (2018). Insulator-to-metallic spin-filtering in 2d-magnetic tunnel junctions based on hexagonal boron nitride. ACS Nano.

[178] Wang WY, Narayan A, Tang L, Dolui K, Liu YW (2015). Spin-valve effect in NiFe/MoS_2_/NiFe junctions. Nano Lett..

[179] Dolui K, Narayan A, Rungger I, Sanvito S (2014). Efficient spin injection and giant magnetoresistance in Fe/MoS_2_/Fe junctions. Phys. Rev. B.

[180] Chen JR, Odenthal PM, Swartz AG, Floyd GC, Wen H, Luo KY, Kawakami RK (2013). Control of Schottky barriers in single layer MoS_2_ transistors with ferromagnetic contacts. Nano Lett..

[181] Iqbal MZ, Iqbal MW, Siddique S, Khan MF, Ramay SM (2016). Room temperature spin valve effect in NiFe/WS_2_/Co junctions. Sci. Rep..

[182] Dankert A, Pashaei P, Kamalakar MV, Gaur APS, Sahoo S (2017). Spin-polarized tunneling through chemical vapor deposited multilayer molybdenum disulfide. ACS Nano.

[183] Wang Z, Sapkota D, Taniguchi T, Watanabe K, Mandrus D, Morpurgo AF (2018). Tunneling spin valves based on Fe_3_GeTe_2_/hBN/Fe_3_GeTe_2_ van der waals heterostructures. Nano Lett..

[184] Karpan VM, Giovannetti G, Khomyakov PA, Talanana M, Starikov AA (2007). Graphite and graphene as perfect spin filters. Phys. Rev. Lett..

[185] Karpan VM, Khomyakov PA, Starikov AA, Giovannetti G, Zwierzycki M (2008). Theoretical prediction of perfect spin filtering at interfaces between close-packed surfaces of Ni or Co and graphite or graphene. Phys. Rev. B.

[186] Cobas ED, van't Erve OMJ, Cheng SF, Culbertson JC, Jernigan GG, Bussman K, Jonker BT (2016). Room-temperature spin filtering in metallic ferromagnet-multilayer graphene-ferromagnet junctions. ACS Nano.

[187] Xie Q, Lin W, Yang B, Shu X, Chen S (2019). Giant enhancements of perpendicular magnetic anisotropy and spin–orbit torque by a MoS_2_ layer. Adv. Mater..

[188] Sbiaa R, Meng H, Piramanayagam SN (2011). Materials with perpendicular magnetic anisotropy for magnetic random access memory. Phys. Status Solidi R.

